# Campus sustainability research: indicators and dimensions to consider for the design and assessment of a sustainable campus

**DOI:** 10.1016/j.heliyon.2022.e11864

**Published:** 2022-11-30

**Authors:** Ayotunde Dawodu, Haoyue Dai, Tong Zou, Hongjie Zhou, Wenhan Lian, Jumoke Oladejo, Felix Osebor

**Affiliations:** a University of Nottingham Ningbo China, 199 Taikang East Road, Ningbo, 315100, China; b University of Grenwich, Old Royal Naval College, Park Row, London SE10 9LS, UK

**Keywords:** Campus sustainability, Sustainability assessment tool, Bibliometric analysis, Higher education, Built environment

## Abstract

Assessment Tools have become the de facto method to address sustainability issues within the built environment. They provide a measurable third-party approach to ensuring sustainability design and directives are met through the use of indictors, criteria’s and credit system. A key challenge is that the existing tools such as BREEAM Communities and LEED Neighbourhood Development address a wider community but cannot efficiently address the more nuanced and context specific sustainability requirements of campuses. However, the approach of utilizing credits, indicator and criteria systems is a tactic that campus planners have slowly began to imbibe. This is even more critical to sustainable development cities due to the huge amounts of land and human resources consumed on campuses; However, gaps exist within the currently existing Campus Sustainability Assessment Tools (CSAT) that have been developed. Generally. Though studies have identified trends and gaps in neighbourhood and building assessment tools, no comprehensive study has investigated the potential gaps from the newly emerging CSATs. Using bibliometric analysis, this study reviews over 1000 articles on campus sustainability and assessment tools with the aim of determining the gaps, trends and focus area of campus sustainability via CSAT. The result draw out 15 dimensions that govern the design of sustainable campuses, and the most predominant dimensions were environmental, educational and governance dimension. The results also highlight the importance of context in implementing and developing CSATs and showed numerous overlooked campus sustainability issues, which have considerable impact on the determining the claims that makes campuses sustainable. Finally, results demonstrated a need to enhance inclusivity in decision making on campus via different survey techniques in both education and implementation of campus sustainability initiatives. In sum this study enhances the development of new CSAT and campus sustainability initiative and the modification existing initiatives to effectively meet the required sustainability standards within the built environment.

## Highlights

• There is a predominant environmental focus of campus sustainability, which is followed by sustainability education and campus governance
• There is a need to address campus sustainability from a more balanced perspective and campus sustainability should not only lean predominantly to the environmental elements
• The one size fit all approach to campuses would be ineffective due to the social, environmental, institutional, and environmental context of each campus
• There is a need to enhance inclusivity in decision making on campus via different survey techniques in education, indicator development and implementation of campus sustainability initiatives

## 1 Introduction

The term “*sustainability in higher education*” was firstly mentioned in the Stockholm Declaration of 1972, bridging the humanity and the environment together and recognizing their interdependency in achieving environmental sustainability (Alshuwaikhat and Abubakar, 2008).

In 2015, the United Nations (UN) established the 17 sustainable development goals (SDGs), as guiding plans for future developments towards the attainment of global sustainability (UN, 2015). Among 17 SDGs, the SDG#4 quality education, SDG#17 Partnership and SDG#11 sustainable cities and communities have emphasized the critical role of Higher Education Institutions (HEIs) as special communities with high potentials for pursuing and promoting sustainable development globally through teaching, research, operations, and knowledge transfer. This has further laid emphasis on the importance of campus sustainability in SD strategies and solutions in many countries.

Velazequez (2006a, pp.812) defines a sustainable university as “*A HEI, as a whole or as a part, that addresses, involves and promotes, on a regional or a global level, the minimization of negative environmental, economic, societal, and health effects generated in the use of their resources in order to fulfill its functions of teaching, research, outreach and partnership, and stewardship in ways to help society make the transition to sustainable lifestyles*” (Alshuwaikhat and Abubakar, 2008). According to Bluhdorn (2017), there are various issues to consider when assessing the sustainability of HEIs, such as environment degradation, biodiversity loss and climate change among many others. Therefore, numerous countries have made efforts to translate these issues into actions and tools through international and national declarations, theoretical and implementation frameworks, assessment tools and evaluation systems to assess the sustainability of HEIs (Shriberg, 2002). This has led to significant strides towards SD (Caeiro et al., 2013). For example, the inclusion of relevant courses focused on green development and environmental sustainability adopted by some HEIs (Roorda, 2002; Lidgren et al., 2006; Lozano, 2009); implementation of environmental management systems on campuses (e.g., ISO 14001 standard, Eco Management and Audit Scheme (EMAS) regulations) (H. M. Alshuwaikhat and Abubakar, 2008; Disterheft et al., 2012; Amaral et al., 2015);, as well as the assessment and publication of sustainability report regularly (Shriberg, 2002; Cole, 2003; H.M. Alshuwaikhat and Abubakar, 2008; Lozano, 2011; Kamal and Asmuss, 2013; Gómez et al., 2014). Other examples can be seen from Universities public declarations on sustainable development (Calder and Clugston, 2003; Wright, 2004; Lozano et al., 2013; Disterheft et al., 2013), specialized conferences are held annually to address and discuss developments in HEI sustainability (e.g., the Association for the Sustainable Development of Higher Education [AASHE], International Sustainable Campus Network [ISCN], and Environment Management for Sustainability in Universities); assessment tools have been developed for assessing campus sustainability (sustainability Assessment Questionnaire (SAQ); Sustainable University Model (SUM); Benchmarking Indicators Questions- Alternative University Appraisal (BIQ-AUA); Unit-based Sustainability Assessment Tool (USAT), and Adaptable Model for Assessing Sustainability (AMAS)), and ranking systems have been established among some universities (e.g, Green Matric; Green League; and STARS).

Hence, multiple research publications in the field of HEI sustainability provide comprehensive overview of sustainability in an HEI context, develop frameworks for assessing the sustainable development efforts of universities, presents insights on best practices for campus sustainability, as well as innovative ways of assessing campus sustainability (i.e., CSATs) (Ramos et al., 2004; Lozano, 2006a; Velazquez et al., 2005; Boer, 2013; Roorda, 2013; Amaral, 2015).

There are currently three main methods of assessing the sustainability of a campus including account assessment, narrative assessment, and indicator-based assessment (See Table 1). Account assessments convert raw data into common units called “accounts” such as monetary, areas, energy etc. This approach is focussed solely on the key aspects of sustainability with limited coverage on some major components of sustainable institutions, thereby limiting its practical utilization (Dalal-Clayton and Bass, 2002). Moreover, issues regarding data availability and scalability further hinders the transparency and accessibility of these tools, making it the least practical method for CSAT. In contrast, narrative assessments usually combine text, map, graphics and tabular data without excess reliance on a specific data type. This attributes to why familiarity and flexibility are the core benefits of narrative assessments. However, its flexibility is partially flawed due to unsystematic and subjective selection of data coupled with unequal treatment which results in coverage gaps and vague priorities. Such limitations of transparency and consistency make narrative assessments ineffective for decision-making, such as strategy development and monitoring. Lastly, indicator-based assessment is one of the most common methods in sustainability evaluation (Du et al., 2020). Despite the diversity of methods and tools for measuring sustainability, indicators tend to play a fundamental role (Ramos, 2009). In order to measure the sustainability of HEIs, many assessment tools are based on indicators. Similar to narrative assessments, indicator-based assessments also make use of text, maps, graphics, and tabular data, but they are organized around systematically developed indicators (Dalal-Clayton and Bass, 2002). One of the helpful features of indicator-based or index-based assessments is their ability to transform various data types into a simplified and meaningful format for various target groups, such as policymakers, decision makers, generally pubic and so on (Ramos and Pires (2013). This makes it more comprehensive, reliable and representative compared to other assessment methods as it is measurable and comparable (Dalal-Clayton and Bass, 2002; Lozano, 2006b). Comparison between the three main approaches for measuring and analysing sustainability in universities is shown below in Table 1.

**Table 1:** The three main approaches to measuring and analysing sustainability.

Approaches	Accounts	Narrative assessments	Indicator-based assessments
Potential for transparency	Low	Medium	High
Potential for consistency	High	Low	High
Potential for participation	Low	High	Medium
Usefulness for decision-making	Medium	Medium	High

Due to their unique benefits of transparency, consistency, participation, and decision-making, this study is focussed on investigating indicator-based assessment tools for developing CSAT.

Though studies have identified trends and gaps in neighbourhood and building assessment tools, no comprehensive study has investigated the potential gaps from the newly emerging CSATs and campus sustainability initiatives a whole. This is even more critical to sustainable development of cities due to the huge amounts of land and human resources consumed on campuses. Furthermore, for assessing sustainability of HEIs’ campuses, indicator-based assessment remains the most suitable and optimal method. Yet, there is currently no universally recognized indicator-based assessment tool for CSAT or an exhaustive set of indicators focused on various sustainability issues. Likewise, most literature reviews and/or indicator banks/indexes/tools on CSATs only cover a few elements of sustainability and don’t investigate the entirety of the potential issues on campuses. Apart from these, existing studies tend to focus on a few isolated tools (i.e., research on one or two tools), which limits the quality of observations, recommendation and mitigation strategies for campus sustainability related issues. Finally, there are limited publications or discussions on the gaps within campus sustainability via the context of assessment tools. There are also limited studies that utilize the Bibliometric approach as a form of investigation into where these gaps and issues can be identified and categorized.

To address these gaps, using bibliometric analysis, this study reviews over 2000 articles on campus sustainability and assessment tools to determine the gaps, trends, and focus area of campus sustainability via CSATs. Also, there will be an analysis of as many CSATs as is discovered which is estimated to be over 10 CSATs. Previous studies have only analysed 1 or 2 CSATs. This will enhance the quality of results due to the larger sample size. The follow up will then be to draw out the common campus sustainability issues and associated sustainability indicators as well as to determine their impacts, relevance, or absence in how sustainability on campuses are achieved. These observations, gaps and trends will then be used to enhance the development of new CSAT and campus sustainability initiative as a whole and provide insight towards the pitfalls of existing campus sustainability initiatives that can to lead their modification thereby contributing the sustainable development of cities.

The materials and methods utilised for this research, the preliminary results of the review, and the summaries of the main findings with future recommendations are reported in sections 2- 4 respectively.

## 2 Materials and methods

**Figure 1:**
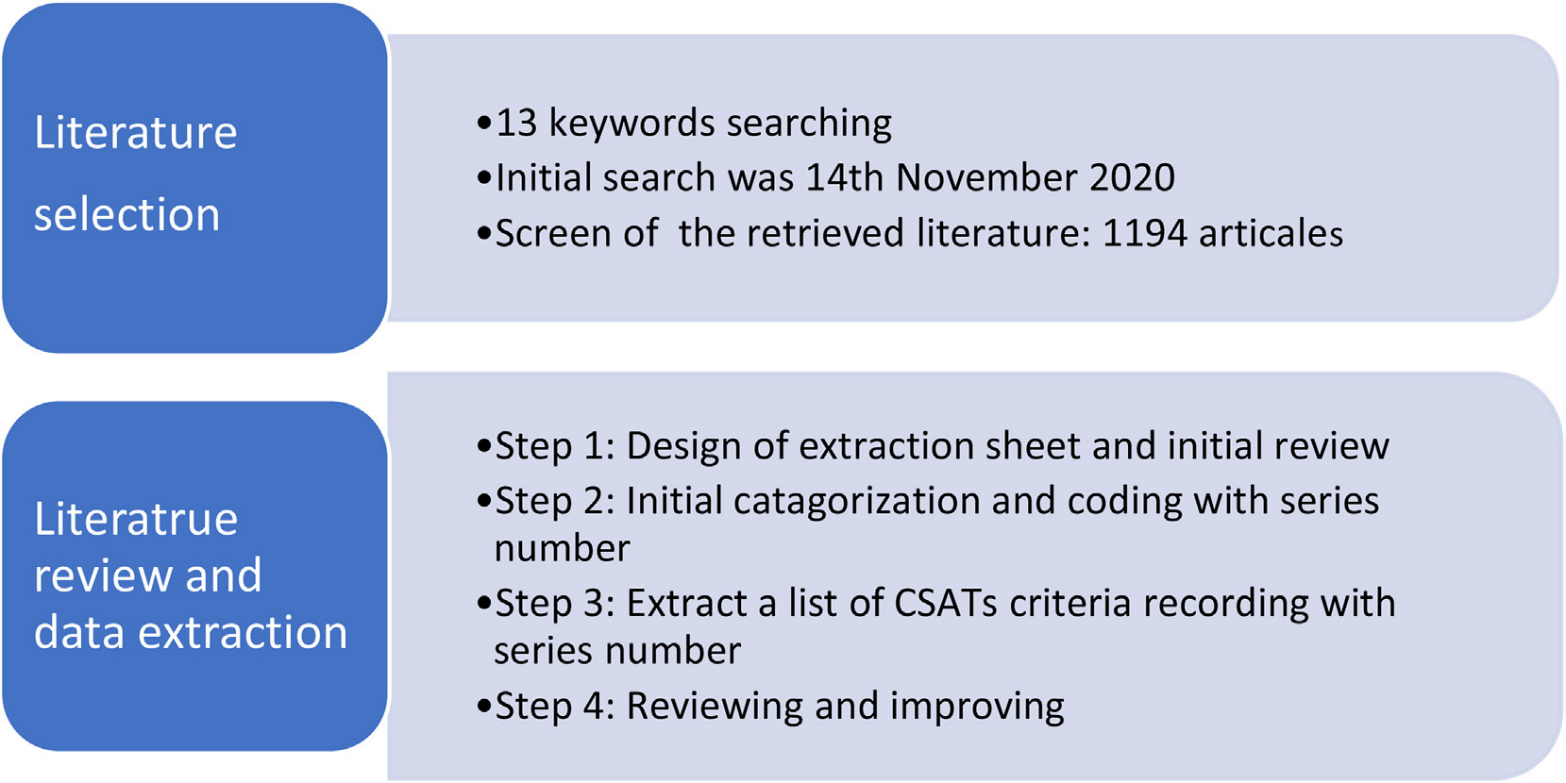
Process of literature review.

Figure 1 depicts a summary of the procedures for literature selection and data extraction which are further explained below.

### 2.1 Keywords selection

In order to source for literatures about CSAT, search strings were formed using relevant keywords. Since this paper aims to study the types, importance, and basis of scoring in existing CSATs, there were some primary and secondary keywords to be selected as searching target. The primary keywords had a higher level of relation with campus sustainability assessment tools compared with secondary keywords. After searching all the relative keywords in Web of Science, 13 keywords were selected to aid the literature. These keywords created for this study include: University Campus and Sustainability’, ‘University Campus and Sustainability Assessment’, ‘University Campus and Sustainability Assessment Tools’, ‘University Campus and Sustainability Certification’, ‘University Campus and Sustainability Campus Tools’, ‘Green Campus; Green Campus and Assessment Tools’, ‘Green Campus and Assessment Tools’, ‘Green Campus and Certification Tools’, ‘Green Campus and Certification’, ‘Green Campus Certification Tools’, ‘Campus and sustainability’, ‘Campus and Sustainability Assessment’ and ‘Campus and Sustainability Assessment Tools’.

### 2.2 Literature selection

The literature research process was conducted using Web of Science due to its extensive database of publications and citations. The Initial search with key words stated in section 2.1 was conducted on the 14^th^ November 2020 and ended 23^rd^ November 2020 using the 13 keywords and a total of 1194 articles without repetition were obtained.

### 2.3 Literature review and data extraction

Data related to sustainability issues and associated indicators were extracted in a three-stage process as shown in Figure 1. Firstly, the list of retrieved articles were compiled into an excel spreadsheet with data related to each article such as the name, location, policies and incentives offered by the university, details relevant to various sustainability categories, government policy, green campus assessment tools, and the stakeholders. Next, the collected data was carefully read and coded with serial numbers by the authors. Then, a list of CSATs criteria were extracted from the spreadsheet which was then related to various components of the assessment tool framework. Based on these extractions, another excel spreadsheet was created for data entry where rows represent the individual criteria extracted from the literature and columns is the corresponding series number which represent the source of this criteria. Finally, another round of review was done for checking and refining through the data for accuracy and completeness of the data entry from Step 3.

### 2.4 Sustainability category selection

Du et al., (2020) highlighted five main dimensions in sustainability within HEIs. These dimensions include governance, operations, education, research and engagement. The operation dimension contains three aspects as environment, social and financial. Similarly, the engagement dimension is subdivided into campus and public aspects. This provides 12 categories which are selected for further analysis (Table 2).

**Table 2:** The selected 12 categories of campus sustainabitiliy

Number	Category/dimension	Defnition/description
1	Governance	Governance dimension includes the vision implemention and management of campus, internal and external community or policy, gender equality, staff hiring, promotion coordination and report assurance process and procedures.
2	Operations-environmental	Operations-environmental dimension refers to the strategies and space utilizations within campus, such as environment auditing expenses and fines, asset and facility, land use, green infrastruture and renewable technologies.
3	Water	Water dimension is the summary of water consumption, water conservation measures, potable water and water recycling.
4	Waste	Waste dimension focuses on the hazardous waste production, waste management and renovation.
5	Building	Building dimension defines building envelop properties and the building functions’ distribution including office, lab, IT, hospital.
6	Transportation	Transportation dimension refers to vehicles, public transportation circulation design and parking system.
7	Operations-social	Operational-social dimension includes working and living circumstances, human rights and staff and students.
8	Operations-financial	Operations-fiancial dimension refers to the actions about sustainability development investment.
9	Education dimension	Education dimension considers about students sustainability education and staff sustainability training.
10	Research	Research dimension includes sustainable research, the support of sustainable research and pulications and implementations.
11	Engagement-campus	Engagement-campus dimension refers to public engagement and sustainable activities, such as studetn’' and staff’' opportunities to work on sustainability and student and staff organizations.
12	Survey	Survey dimension defines the research related to sustainable survey to staff and student.

## 3 Results and discussions

### 3.1 Background on campus sustainability assessment tool (CSAT)

According to the results from the literature review as shown in Table 2 and 15 CSATs have been developed between 2006 and 2019. Green Metrix (GM), the first CSAT was established in 2006, where scoring of sustainability agenda or issues was based on response rate. After that, four more improved assessment tools were proposed in 2019, including the new Green Metrix (GM), People & Planet Green League (P&P), Sustainability Tracking, Assessment and Rating System for Colleges and Universities (STARS), and the Assessment Standard for Green Campus (ASGC). Other assessment tools released from 2009 to 2014 include Assessment System for Sustainable Campus (ASSC), Graphical Assessment of Sustainability in University (GASU), Greening Universities Toolkit (Toolkit) etc. It is worth noting that 9 out of the 15 CSATs included were proposed after 2013, three from 2013, one from 2014, one from 2016, and four from 2019. Overall, a third of the CSATs were developed after the SDGs were established in 2015 which is an indication that the growing trends and global recognition for campuses sustainability is related to the drive for global sustainability. Similar trends have been found with the use of assessment tools and their associated indicators as a methodological approach for guiding various sustainability initiatives.

In terms of citation frequency, the most popular CSAT identified by this study is GASU which was developed in 2011, with mentions from 174 studies. The ASSC built by Sustainable Campus Management Office of Hokkaido University in 2013 comes second with 170 citations; followed by Greening Universities Toolkit from 2013 with 134 mentions, Pacific Sustainability Index from 2011 (83 times), and Sustainability in Higher Education Institutions (SusHEI) developed in 2013 had the lowest mentions with only 16 mentions as illustrated in Table 3.

**Table 3:** CSATs included in the reviewed literature.

No	Tool	Main developer(s)	Origin	Year	Citation Count
1	Assessment Instrument for Sustainability in Higher Education (AISHE)	Dutch Foundation for Sustainable Higher Education	Global	2009	30
2	Adaptable Model for Assessing Sustainability in Higher Education (AMAS)	Francisco Urquiza Gómez et al. (2014)	Chile	2014	25
3	Assessment System for Sustainable Campus (ASSC)	Sustainable Campus Management Office of Hokkaido University	Japan	2013	170
4	Campus Sustainability Assessment Framework Core (CSAF Core)	Sierra Youth Coalition (SYC)	Canada	2009	48
5	Graphical Assessment of Sustainability in University (GASU)	Global Reporting Initiative	Global	2011	174
6	Green Metric World University Rankings (GM)	University of Indonesia	Global	2019	39
7	People & Planet Green League (P&P)	People & Planet	UK	2019	69
8	Pacific Sustainability Index (PSI)	Roberts Environmental Center of Claremont McKenna College	USA	2011	83
9	Sustainability Assessment Questionnaire (SAQ)	University Leaders for a Sustainable Future	Global	2009	25
10	Sustainability Tracking, Assessment and Rating System for Colleges and Universities (STARS)	Association for the Advancement of Sustainability in Higher Education	North America	2019	69
11	Sustainable University Model (SUM)	80 HEIs	Global	2016	27
12	Sustainability in Higher Education Institutions (SusHEI)	HEIs	Portugal	2013	16
13	Greening Universities Toolkit (Toolkit)	Africa, Asia-pacific, Europe, Latin America, and North American universities	Global	2013	134
14	Unit-based Sustainability Assessment Tool (USAT)	Swedish/Africa International Training Program	Swedish/Africa	2009	75
15	Assessment Standard for Green Campus (ASGC)	Chinese Society for Urban Studies	China	2019	57

In terms of geographic origin, the investigated CSATs mainly originated from nine countries/regions (Chile, Japan, Canada, UK, USA, North America, Portugal, Swedish, and China) indicating the importance of the research on campus sustainability globally. Yet, it is observed that most of these CSATs come from North America, Europe, and East Asia while only a few have been referenced from South America, and other developing/underdeveloped countries where great importance should be attached to sustainability. For example, Chile is the only South American country with research on campus sustainability, and other regions such as African and South Asia currently do not have any research on campus sustainability. The results also imply that developing countries like Chile and China have only recently started research on campus sustainable development, and started their CSATs development in 2013 and 2019 respectively.

Table 3 provides a summary of the tools that appeared in the 1194 articles and the number of occurrences is counted in the last columns. Note that the following table only represents publications within the scope of the research methods.

**Figure 2:**
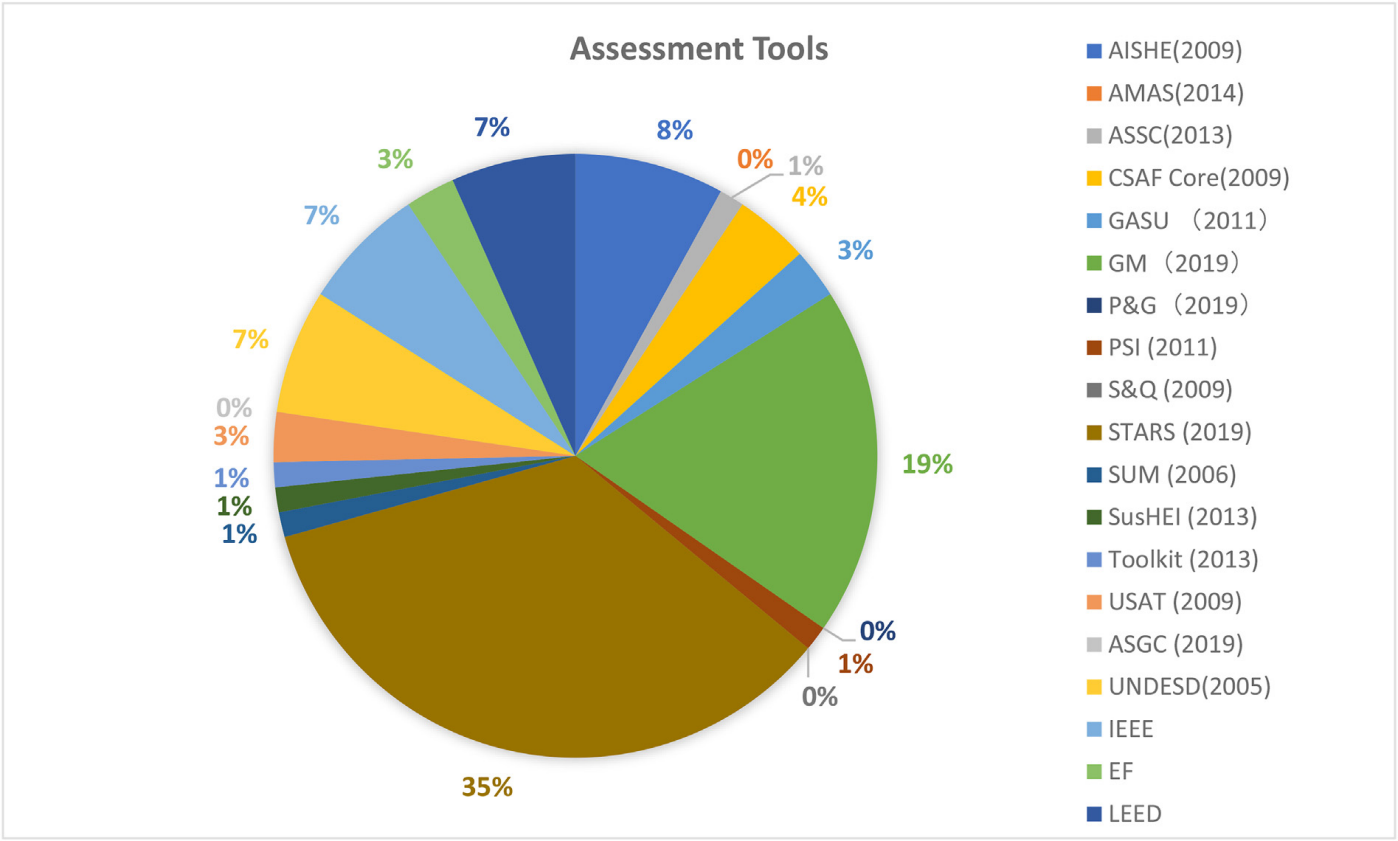
Percentage distribution between assessment tools occurrence in reviewed literature.

Figure 2 illustrates the frequency distribution of various CSATs studied in the 1194 articles. Besides the 15 CSATs previously mentioned in Tables 3 and 4 additional assessments - UNDESD (UN decade on education for sustainable development) (2005), IEEE (Institute of Electrical and Electronics Engineers), EF (Environmental Footprint), LEED (Leadership in Energy and Environmental) - have also been considered in Figure 2 in the analysis. These additional considerations were through snowball literature sampling. This mean that these tools were noted to have been used in some capacity to effect sustainability change in a systematic manner within campuses. In detail, STARS has the biggest frequency rate of 35%, followed by GM with 19%, AISHE with 8%, and UNDESD, IEEE, and LEED rank the same as the fourth highest with 7% frequency rate. A key reason for STARs popularity could be the fact that the tool is a spin-off of the already popular STARs community and has shown successful market penetration. Also, the American region is noted to focus significant amount of its research capacity on assessments tools as compared to other countries (Dawodu et al., 2022a). Other tools only have very weak frequency of occurrence under 5%, while AMAS, P&G, S&Q, and ASGC have no occurrence at all within literature during the time investigated.

**Table 4:** Results and recommendations for future CSATs.

#	Dimension/topic	Positive	Negative	Recommendation
1	Governance (9%)	Policies in the governance (36%) dimension provide guidance for sustainable development; HEIs’ campuses are important test beds and leadership roles for various types of sustainable solutions	Gender equality (1%), staff enterprise hiring and promotion coordination (1%), and reporting assurance process and realization (1%) are less common; there is a gap between students and the application of university sustainability programs	Improve areas of socio-institutional aspects that govern equality, fairness etc; sustainability policies need to be tailored to the context of the campus; increase its percentage contribution among the other 11 categories by addressing the 7 criteria as comprehensively as possible.
2	Operations-environmental (30%)	Energy is the current focus of operations-environmental SD for HEIs’ campuses (i.e., energy consumption [20%], energy efficiency measures [16%], renewable energy [8%]; greenhouse gas emissions [17%], greenhouse gas reduction measures strategy [8%]); great variety of indicators in this topic involving land use, ecosystem, policy system and even contracts and purchase products and services	Pesticides (0%), safe sites (0%, and hospital plan (0%) have been ignored; the concept of examining environmental management performance (EMP) in the context of higher education remains non-existent	More considerations of the ecological aspect of operations-environmental topics; supportive infrastructures, implementation planning, and land use considerations are also suggested
3	Water (6%)	Only four aspects are covered and distributed relatively even; water recycling/reuse strategy (32%), consumption (30%) and conservation measures (28%) are well documented	Lack of consideration for water supply facilities and technologies, water cost/price, implemented policy and strategies, water quality/hygiene level (0%)	Cover the lacking aspects; other types of natural resource like land, biodiversity etc.
4	Waste (6%)	Four aspects have been covered, the least popular aspect is indoor environment (11%); the most popular aspect is waste system design, construction, renovation (34%), followed by waste system maintance and operation (33%), and total amount hazardous waste (22%).	Lack of waste management standards/policies, waste disposal methods, cost of each type of wastes (0%)	More consideration of indoor environments, wastes generation, waste types and cost, as well as waste disposal methods.
5	Building (4%)	Five aspects have been involved in building topics, the most popular is strategy (46%), followed by material (27%); historical buildings are considered but with only a very small proportion (3%)	No green office (0%) is covered in building topic, green lab (12%), green IT (12%) are limited considered.	All the green aspects could be merged as one; building utility rate and longevity, building land use, building types, functions and distributions are suggested to be considered in the future CSATs
6	Transportation (6%)	Five aspects are found in literature related to transportation topic, vehicles (45%) and public transportation and circulation design (34%) are most concerned	Limited consideration of commute model split (9%), parking (7%), and slow traffic (5%).	Transportation-related pollution, incentives, and policies, governmental supports and encouragement are recommended
7	Operations-social (6%)	Only three aspects belong to this topic and working and living circumstance (56%) is the dominant interest of research. More specifically, the top three peformers under working and living circumstances are students’ affordability and access to education (32%), smart tools (26%), safe, fair and healthy circumstances (19%)	Human rights of staffs and students (29%); social and environmental responsibility (15%) are limited in consideration	May also consider aspects more specific such as students’ social activities; social satisfactory rate; mental health considerations may also reflect some images of HEIs’ operation-social dimensions
8	Operations-financial (3%)	Six aspects are covered in this topic, economy performance (63%) occupies more than 50%, followed by budget, expenses and investment (19%)	Very limited consideration on funds for operation (3%), funds, revenues for research (3%), purchase, supply chains (6%), environment and social health and safety fines (6%); lack of consideration for strategies for operation (0%), tuition fees (0%), wage gap (0%), local development investment (0%)	Besides those limited aspects, tuition fees, wage gap, local development investment are also recommended
9	Education (17%)	Only two aspects are observed while students sustainability education accounts for 91% in the literature. In detail, four subtopics are covered in students’ sustainability education: plan (36%), curriculum (35%), supports for curriculum programs, experiences learning skills (15%), literacy assessment (14%)	There is lack of consideration for staff sustainability training (9%); limited consideration of supports for curriculum programs, experiences as learning skills; and literacy assessment for students sustainability education	Future directions may involve development of a systematic training procedure or standards, inventing some certifications of training qualification, selection of training modes and contents, clarifying HEI and staffs’ roles and responsibilities as regards sustainability
10	Research (7%)	Majority of studies covered sustainable research (94%)	Lack of consideration for implementation (0%) and very limited coverage on research supports (6%)	Increase the coverage of research plan, research integrating SD issues, research facilities, skills training, collaboration,
11	Engagement-campus (2%)	Only two aspects have been covered in this topic, activities occupies the most proportion (85%) while public engagement accounts for 15%. For activities, most considered topics are programs (29%), students’ and staffs; opportunities to work on sustainability (29%), incentives information and communication evaluation (18%), students and staff career development (12%); the rest are below 10%	Limited consideration for students and staffs organization and orientation; very limited coverage of public engagement related aspects (i.e., volunteer service, campaigns, programs and partnerships	Improve Forms of engagement, enhance strategies for people’s willingness to participate, improve HEI’s reputation on engagement activities, budgets and strategies as well as feedbacks may also need to be considered; moreover combining top-down and bottom-up approaches together is recommended
12	Survey (4%)	There are 5 topics included in the survey topic, views about sustainability attitude, awareness, challenge and benefit have taken place 47% of literature	Subtle coverage on food (16%), education (18%), sustainability development (16%); very limited consideration of wastes recycle (3%)	Improved the less considered aspects in future research; and involve other elements like different form of campus sustainability research

### 3.2 Current focus of CSATs

**Figure 3:**
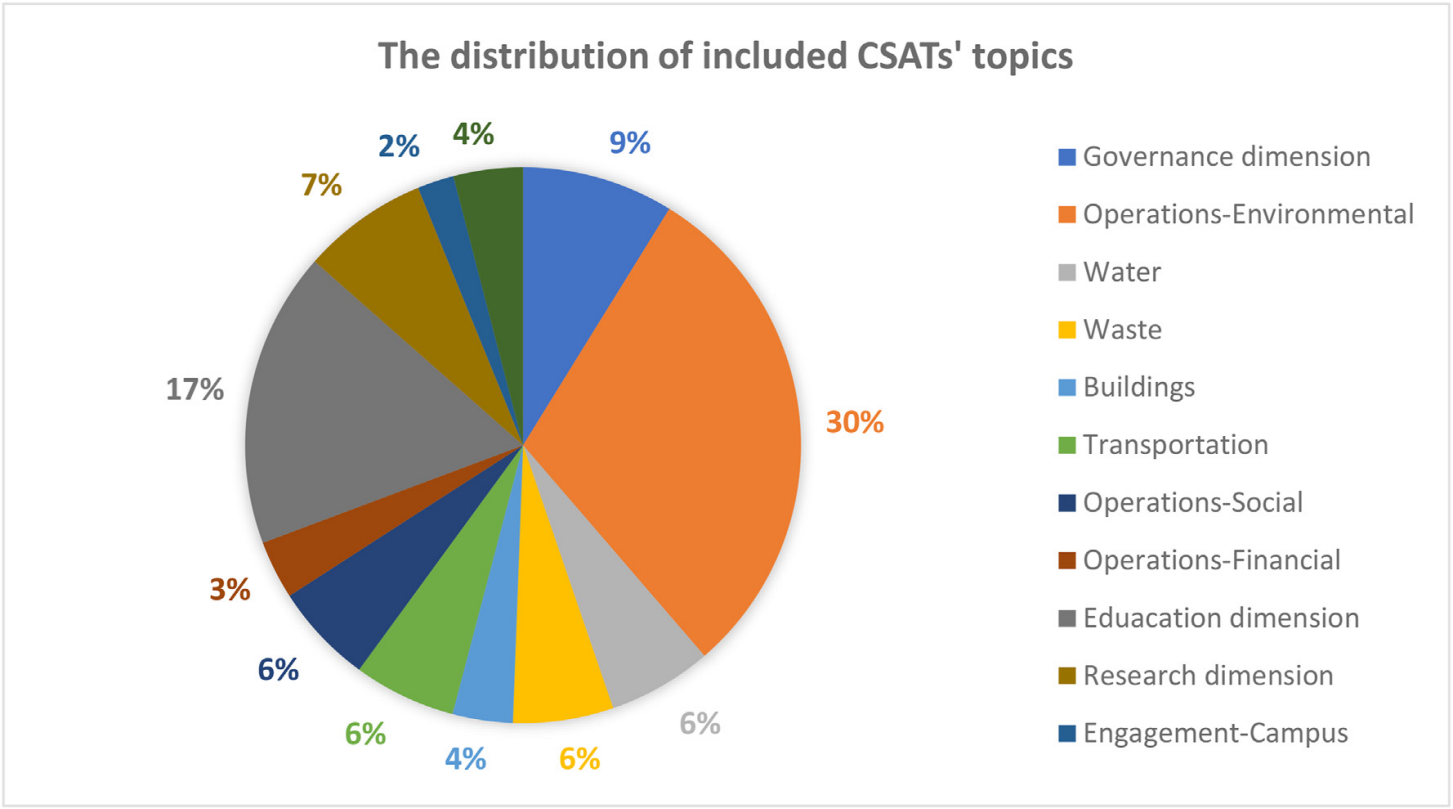
The distribution of included CSATs’ topics.

**Figure 4:**
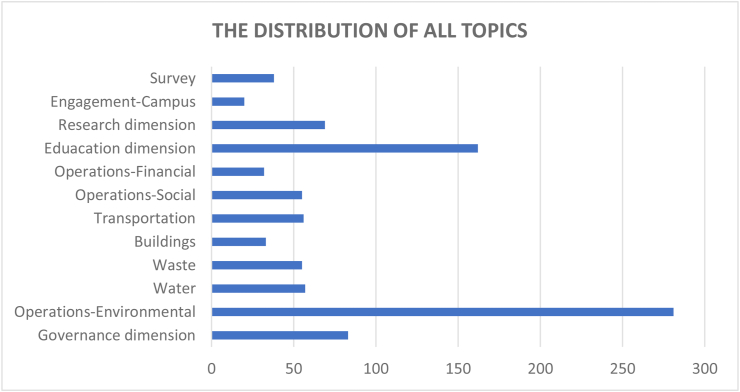
The distribution of included CSATs topics.

The evaluation criteria utilised by the 15 CSATs previously listed in Table 2 are evaluated and divided based on the 12 sustainability categories for in-depth analysis. The categories are governance dimension, operations-environmental, water, waste, buildings, transportation, operations-social, operations-financial, education dimension, research dimension, engagement campus and survey. Figures 3 and 4 show the distribution of these categories across the accessed papers by investigating how many literatures have mentioned these topics. This was done in order to study the importance of various sustainability categories to the developed CSATs. From these two figures, it is observed that the two topics with the highest relevance are operations environmental (30%) and education dimension (17%) indicating their importance to CSAT research and invariably campus design and development. Closely followed by governance dimension (9%), research dimension (7%), water, waste, transportation, and operations-social (6%) and so on. The large disparity between the considerations of these categories could be an indication of a expected focus of current CSATs. This is because a frequently identified gap of assessment tools is their emphasis on the environmental aspect of sustainability (Dawodu et al., 2022a; Onyango and Adewumi, 2021), likewise an expected outcome of a campus sustainability tool would be the focus on sustainable education or education or sustainable development.

There is a consensus in literature that a sustainable university campus results needs a better balance of the economic, social, and environmental objectives in policymaking, as well as a long-term understanding of various campus activities (Di Gerio et al., 2020). However, the reason why some categories are more relevant while others are sparsely considered remains unclear in literature. In order to explore potential reasons for this, Section 3.3 discusses the advantages and limitations associated with current CSATs with reference to the 12 dimensions.

### 3.3 Understanding the advantages and limitations to CSAT focus

Here, 12 sustainability dimensions of the CSATs will be assessed in detail to reveal their benefits and limitations.

#### 3.3.1 Governance dimension (83 papers)

**Figure 5:**
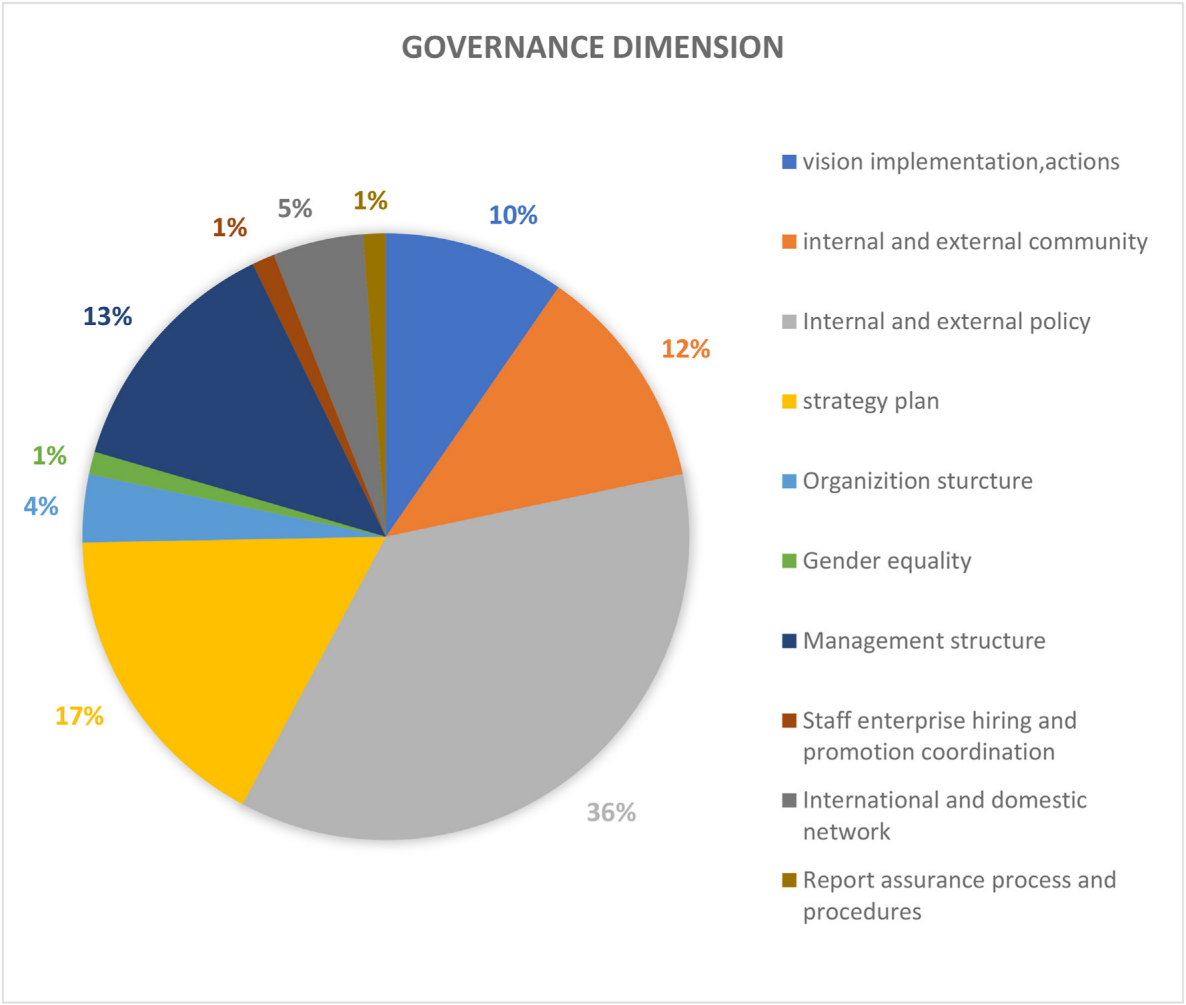
Coverage on the topic of governance dimension.

In total, 83 papers covered the governance dimension in Figure 5. The governance dimension refers to a sustainable development policy in terms of the HEI's policy structure. While these policies and structures are abstract, the main essence of governance dimension is closely related to the structure of HEIs and campus norms. This includes who and/or how power is exercised for the development of the environment and the rights and obligations of individuals within the HEI and campus. Based on the review articles, governance dimension can be subdivided into 3 main aspects, policies, economics, and the institutional aspects. Policies are guidance and guidelines for sustainable development. The aspects of internal and external policies (36%), strategic plans (17%), management structure (13%), internal and external groups (12%) ，vision implementation and action (10%) account for a significant proportion of governance dimension as seen in Figure 5. As these aspects are all at the macro level, they constitute key elements at the core of the campus and reflecting the university's role as an agent of social change to provide knowledge, innovation, and solutions to society by cultivating sustainable talent. Universities are therefore uniquely positioned to assist in the implementation of the 17 SDGS and its 169 targets for ultimately promoting economic prosperity, social inclusion, and environmental sustainability. Sustainable university transformation can be achieved through four key management elements: value, strategy, partnership, and transparency (Di Gerio et al., 2020). Apart from these four management elements, HEIs can also rely on scientific frameworks for sustainable development to restructure their administrative structures. For example, the 6-P framework of community participation which includes psychological needs, physical facilities, personal motivation, public perception, price mechanism and policy was determined to be highly adaptable to university communities when a case study was conducted for two universities (Too and Bajrachary, 2015). Monash University carried out four activities to promote sustainable lifestyle among the campus community. The activities were Monash Footprints, Bin There, Done That, Monash Environmental Pledge, and Sustainable Transport Fiesta. Monash Footprints engaged participants in various hands-on activities for cooking sustainable food, promoting organic and fair-trade products and reducing their daily environmental footprint. 'Bin There, Done That' was established to increase the waste collection and increase awareness about appropriate waste disposal strategies. Similarly, the Monash environmental Pledge and sustainable transport fiesta were implemented to further declare their commitment to sustainability in terms of energy, waste, food, water and transportation by installed green facilities and promoting sustainable transport options. Apart from Monash, Cornell University also conducted four successful sustainability projects using the 6-P framework named Green Teams, Lights Off Cornell, Take Back the Tap and Public Transport Service. To achieve this, they incorporated sustainability into department's daily operation, encouraged energy conservation practices, reinvested in public water infrastructures such as water filling stations, and subsidized regional public transportation costs. The project was estimated to save up to $60,000 a year.

Alternatively, various societal policies are often piloted on campuses before being implemented on more practical and broader applications. Essentially, making HEIs campuses pilot studies for future city-wide sustainability initiatives and an important test bed for various types of sustainable solutions (White, 2020). In sum, the transition to sustainable development cannot be achieved without social innovation, whether on HEIs’ campuses or elsewhere (Fraga-Lamas et al., 2019). In line with this, studies by Zint (2016) and Zou (2022) further highlight the scope at which universities play a strong leadership role in promoting and supporting sustainable food systems through integrated institutional policy and governance mechanisms.

A key limitation of the governance dimension is the underrepresentation of some aspects such as gender equality, hiring and coordination etc which are still at the initial stage of sustainable practice on campuses globally. Hence, less occurring issues such as gender equality (1%), staff enterprise hiring and promotion coordination (1%), and reporting assurance process and realization (1%) were hardly discussed in the reviewed articles. This, in addition to general bias towards the environmental and economic routes of sustainability on campus can create a blind spot and further shift focus away from weaker but still relevant governance issues (Lozano, 2006a; Newman, 2006; Velazquez et al., 2006; Lopes & Thiago, 2021).

The pathway to a solution that optimizes governance dimension is not straight forward, as it is difficult for HEIs to choose an ideal sustainable practice mode of operation due to context, finances, geographic location, external factors such as legislative policies, technological capabilities and university reputation etc (Xiong and Mok, 2020). However, the consensus is that environmental and economic measures must also consider social and human factors, otherwise the whole premise of sustainability is defeated (Anwar et al., 2020). Accordingly, universities must be inclusive, actively involved, and interactive with all concerned parties (Bayas Aldaz et al., 2020).

Moreover, if sustainability is to be achieved, then it should be enforced and implemented throughout the entire university structure while considering influential and less influential stakeholders. Students happen fall under the category of influential stakeholders, yet they are impacted the most by campus operations, yet, previous studies have indicated a gap between students and the application of university sustainability programs (Ulkhaq et al., 2018). Therefore, it can be argued that solely relying on perceived propaganda from universities’ administrative departments for implementing an effective sustainability campaign is not adequate. Instead, it is essential that such programs must be implemented to improving individuals’ awareness and consciousness of campus sustainability.

Overall, the governance dimension of sustainable development needs to be improved on campuses due to the identified gaps in implementation and within the socio-institutional aspects that govern factors such as equality, fairness etc. In addition, sustainability policies need to be tailored to the context of the campus, and the results should not be generalized and compared to other campus scenarios (Fraga-Lamas et al., 2019). Consequently, it is suggested that the percentage contribution of the institutional dimension (8%, see figure) should be increased in comparison to other dimensions by addressing the ten criteria’s as comprehensively as possible by optimizing both campus design and CSAT criteria, indicators and credit allocations.

#### 3.3.2 Operations-environmental dimension (281 papers)

In 2019, non-renewable energy accounted for 84% of the total energy demand globally (Mufutau Opeyemi, B., 2021). Such energy sources are characterized by high carbon emissions, resource depletion, and its negative impact on the environment, which cannot be ignored （Pardo et al., 2019). Educational institutions are one of the largest public sectors in many countries, and the size of many campuses are equivalent to small cities （Chakraborty et al. (2021). For example, in China, the education sector is the largest public sector and consumes 40% of the total energy within the public sector. Similarly, HEIs in Canada typically require 60% of the electricity allocated to the education sector, equivalent to a small city of about 400,000–450,000 homes (Alghamdi et al., 2020). Due to the impact of ‘energy’ in improving global environmental and economic situation and the scale of consumption in HEIs, most research articles on campus sustainability are focused on energy issues (Abdul-Azeez & Ho, 2015).

**Figure 6:**
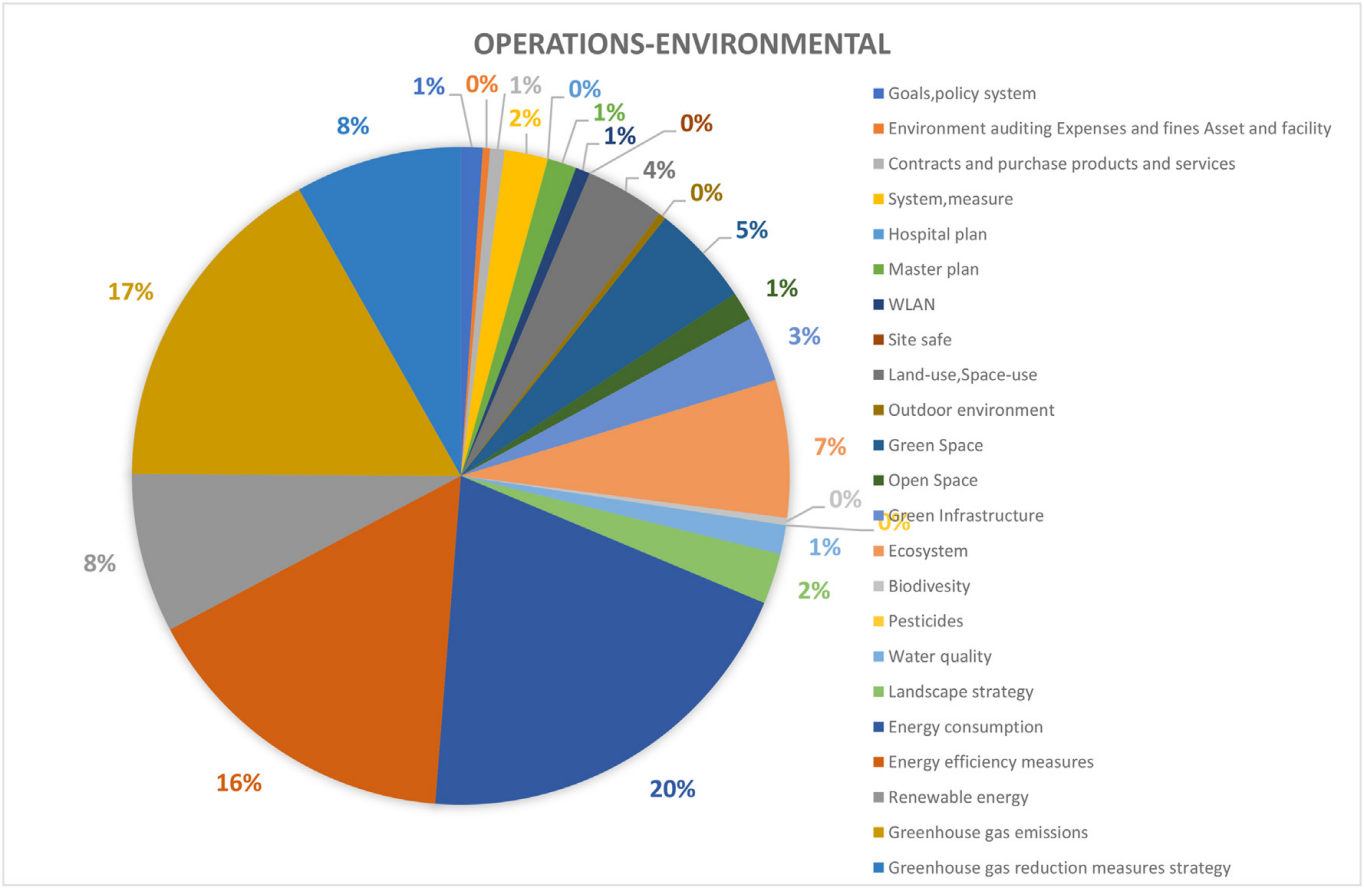
Coverage on the topic of operations-environmental.

**Figure 7:**
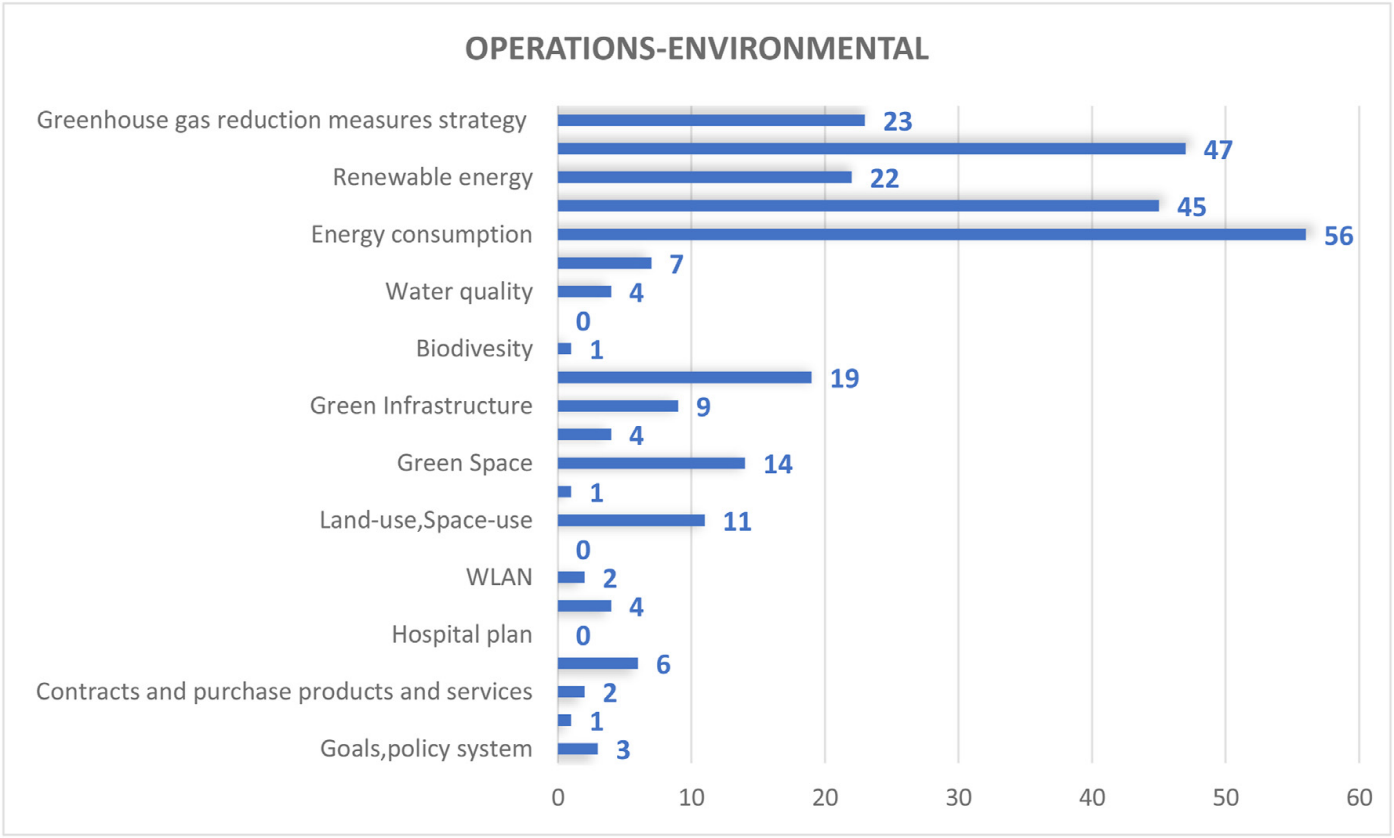
Coverage on the topic of operations-environmental.

Overall, 281 studies covered the operations - environmental dimension of CSATs. As shown in Figures 6 and 7, energy consumption (20%), greenhouse gas emissions (17%) and energy efficiency Measures (16%) were the most considered aspect in this dimension with the obvious commonality that they are all directly related to energy use. Energy consumption is a key focus of campus research due to the multidimensional impact of energy consumption, which affects both the environment, financial operations, and social activities of any campus. Comparably, greenhouse gas emissions is also crucial due to its impact on global air quality, health-related implications, and contributions to global warming. In context, most activity on campuses incur electrical energy demand such as lighting and refrigeration and is inextricably linked to greenhouse gas emissions and fossil fuels (Lukman, 2009). This makes reduction of energy use by implementing energy conservation strategies and adopting energy efficient facilities important, as exemplified by the third considered aspect in this dimension. Therefore, it is imperative to operate campus buildings and equipment in an energy-efficient manner and to take saving measures wherever possible to simultaneously reduce energy consumption and associated greenhouse gas emissions (Simpson, 2003).

The next criteria in this dimension are Greenhouse Gas Reduction Measures Strategy (8%) and Renewable Energy (8%), both of which are energy subsets. This high focus on energy for promoting environmental sustainability is mainly associated with the current dependency on polluting energy sources such as fossil fuels. In contrast, some aspects such as pesticides, safe sites and hospital plan, which are more ecological in nature have not been well considered. Oddly, these aspects actually have more to do with local air quality and emissions surrounding the construction and planning of the campus environments and its residents. Biodiversity, outdoor environment and environment auditing assets and facilities each account for only 1%. This is also corroborated by Kermath (2007), who explained that biodiversity often takes a back seat in comparison to energy use, resource consumption and waste management in the field of campus sustainability. Comparably, the study by Tsai et al. (2012) stated that environmental management performance (EMP) checks play a little role in assessing the ecological sustainability of higher education institutions. This highlights the need for HEIs to expand their responsibilities in dealing with ecological sustainability from the operational level to include larger aspects of management functions. Still, the concept of EMPs in the context of higher education remains non-existent (Jarillo et al., 2019). Consequently, the analysis of environmental problems is a key concern in the sustainable design and operations of campuses.

In general, many sustainability measures are still lacking within this dimension. For example, the usage of renewable energy in most campuses do not match the popularity level of what has been taught and/or publicized in most universities. This means that universities teach and undertake research in renewable technology frequently but rarely implement them within campuses and the main energy source for campus operations still come from traditional fossil fuels. The lack of high scale implementation can be attributed to renewable energy systems requiring higher capital costs, better supportive infrastructures, more significant planning, and land use considerations than traditional energy, as well as their low utilization efficiency. To sum up, HEIs operational-environmental issues need to consider both indirect and direct consequences of energy use and the ideal methods to address them. HEIs need to move one step forward from simply teaching alternate energy options to implementing renewable energy application on campuses. Also, it can be concluded that exploring more cost-effective ways to implement renewable energy on campus level should be one of the key focuses of campus sustainability research as well as the operational environmental aspects of HEIs.

#### 3.3.3 Water dimension (57 papers)

Since the 20^th^ century, there has been an increasingly rising attention to the global water issues due to the growing climate change impacts, pollution and environmental degradation as a result of population growth and rapid urbanization. The importance of water has been well recognized for human survival, development, and long-term prosperity, hence, its critical role in sustainable development and socio-economic activities.

**Figure 8:**
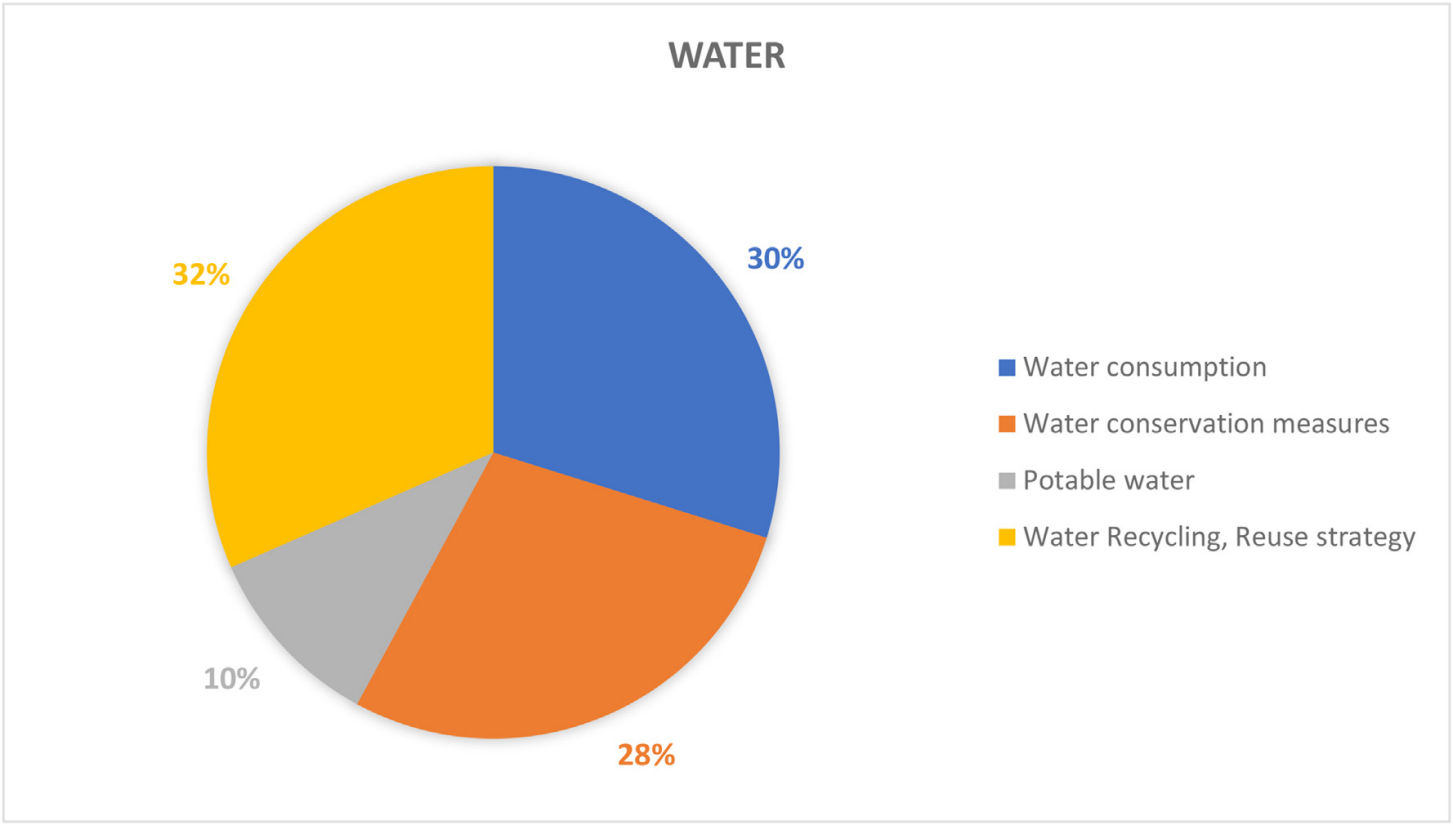
Coverage on the topic of water.

According to Smith et al. (2018), energy used in water treatment accounts for 4% of global electricity consumption which is equivalent to 50 million tons of oil. At the same time, water scarcity is a major problem that does not only an impact water supply management, but also affects drinking water standards and associated economic gains of water supply (Abdul Ghani et al., 2019). HEIs by nature promotes sustainable and integrated water management through education, research, services, and operational activities. In terms of the water resources (Figure 8), there the proportion of recycling strategy (32%), consumption (30%) and protection measures (28%) of water resources are comparable and correlated to building facilities water usage. In terms of water recycling strategies, universities implement different types of water recycling technologies to improve resource utilization and reducing the economic impact on the campus. It is becoming more important to evaluate the operations of universities and the impacts of human behaviour patterns and the environment via an Energy Management System (EMS). A study conducted by Alghamdi et al. (2020) showed the direct impacts, link and interrelationship of electricity consumption and water use. An instance can be seen in the University of Northwest Malaysia (UUM), which spent about $1.2 million USD to meet its water consumption of nearly 4.5 million cubic meters with approximately 15% of water distribution lost every year, which translates to significant of money being lost (Abdul Ghani et al., 2019). Thus, measures needs to be in place on campuses to address water leakages. This can be from both design and installation perspective. For instance, ensuring that pipe design and fittings are at their optimum and utilizing water leakage detection and shut off technologies. Another key aspect of water sustainability on campus is the re-use and recycle measures as shown in Figure 8. For instance, with the aforementioned university, the campus implemented the use of a rainwater collector (RWH) in response to local weather conditions, in which 75% of the rainwater was collected and used for toilets and fire extinguishing systems. Rainwater harvesting has become an important source of water in some areas, especially in urban areas (Silva et al., 2019). Also, University of California Davis uses a permeable pavement as an alternative to asphalt, which improves water sustainability and reduces runoff water treatment (Terhell et al., 2015).

The proportion of potable water (10%) research on campuses is relatively small, underlying two possible reasons. First, existing facilities of clean water and potable water for most HEIs in included studies are already quite good and there is little to do concerning potable water and campus sustainability. Second, the cost of initial investment is relatively high for HEIs to convert non-drinking water into drinking water thus it is within reason that they transfer this burden to municipal water utility companies that are more equipped to handle the technicalities of this endeavor. A noteworthy factor is the influence of the public on the implementation of some sustainability projects (Smith et al., 2018). This was discussed in reference to a famous case in Australia which evolved from a referendum on an indirect drinking water reuse scheme in which more than 60% of voters rejected the proposal, leading to its abandonment. Consequently, it is believed that the public's understanding of a project determines the success or failure of the project, and high public opposition can become major obstacles to the implementation of different green projects, even within campuses (Smith et al., 2018).

In sum, water sustainability and the varying dimensions need to be considered holistically, with context, planning approach, technology and resources of the university in mind. This will help develop the best approach to sustainable water management. For instance, currently Integrated Water Resources Management is a key approach to sustainable water management that enables university campuses address water quantity and quality issues simultaneously. This implies that improving campus water sustainability issue by issue or separately is not comprehensive or effective enough, thus calls for more research and practices employing an integrated and holistic view would be ideal. Universities have several particular characteristics enabling them to become an effective mode of sustainable water resources management and proving ground. These features include considerable autonomy and management of tangible campus landscape, the influence of the multi-level and knowledgeable experts that can help to develop and address integrated water resources management, and opportunity to involve all stakeholders in order to design a more optimum outcome to water issues (McHugh, 2011). Of course, there are certain limitations in the utilization of water resources. Since each HEIs’ campus has its own unique environment, the measure of success in one university may not be applicable and practical in other universities, bringing multiple difficulties in copying and/or benchmarking standardized approaches for improving water resource usage and utility on HEIs’ campuses. In addition, many HEIs have not fully implemented or invested in water-saving measures as most of them are usually emerging technologies with heavy financial investments required and a long payback period.

#### 3.3.4 Waste dimension (55 papers)

**Figure 9:**
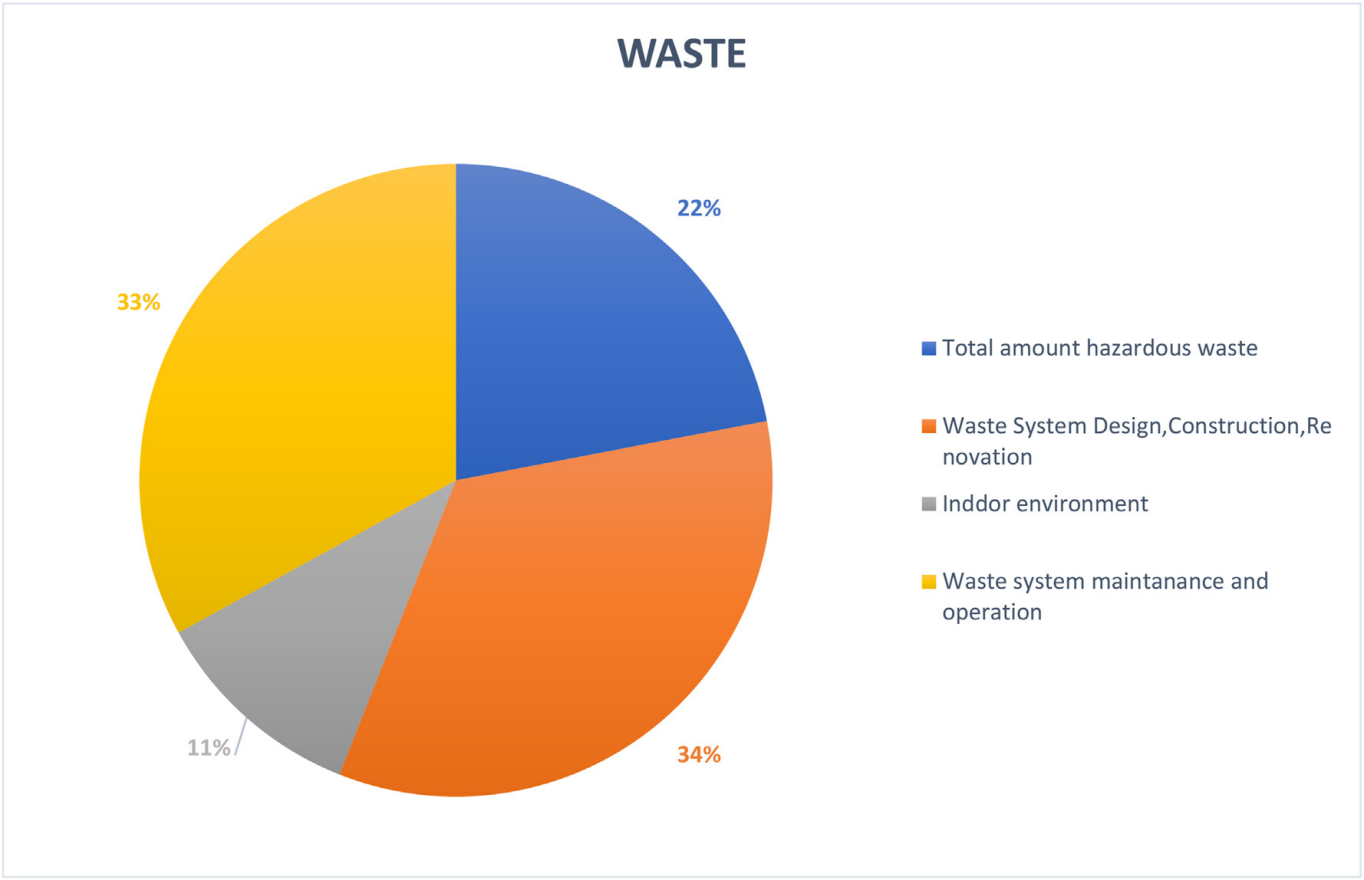
Coverage on the topic of waste.

HEIs are expected to drive social change and promote infrastructural development, including waste management on a small city scale (Tangwanichagapong et al., 2017). However, achieving sustainability with regards to integrated Solid Waste Management (SWM) programs is one of the biggest challenges for HEIs (Smyth et al., 2010). Effective SWM requires an understanding of each component of the waste as well as the source and causes of their generation. Figure 9 shows that waste system design, construction, and renovation (34%) accounted for the largest proportion of waste treatment topic. This element is important in any campus as a waste plans and policies needs to be in place to ensure a sustainable outcome to waste practices. In Europe and parts of the world these designs are governed by European Union (EU) waste hierarchy, which addresses waste in a hierarchical order. This strategy is ranked as follows, waste reduction, waste reuse, waste recycling and composting, waste recovery (Energy) and then landfill. To execute this on any urban or campus scale direct waste characterization is one of the most effective processes for identifying waste types and opportunities for reduction, reuse, recycling, and composting (Tangwanichagapong et al., 2017). Thus, Waste management is not only a de-facto approach that many campuses within the EU already practice but has become non-negotiable agenda for higher education institutions claiming to sustainable and is actively included in campuses in regions such as New Zealand, Japan, and Thailand. In fact, the aforementioned universities have used sustainable waste management practices as a starting point in developing their wide campus sustainability programs (Tangwanichagapong et al., 2017).

In terms of the maintenance and operation of waste systems (33%), introducing sustainable waste initiatives addresses an aspect of sustainable waste utility, however this process which often require heavy machinery, equipment, and transportation, needs to be efficiently maintained. For instance, waste separation practices and operations should be properly implemented by all university members, these elements enhance the waste management process by reducing moisture content in waste. Moisture content can be the sole determinant if adequate energy can be recovered from waste or if the waste composition generated from campus is even tenable for process such as anaerobic digestion, biomass combustion and pyrolysis (Suwartha and Sari, 2013). Apart from the waste systems, there are several studies on hazardous waste (22%), such as electronic waste and solid waste (Saldaña-Durán and Messina-Fernández, 2021). Various studies reveal multiple negative impacts of electronic waste exposure on human health, including change in thyroid function, changes in cellular expression and function, adverse neonatal outcomes, changes in temperament and behaviour, and decreased lung function (Grant et al., 2013). Still, the influence of waste on indoor environment (10%) is not well considered as more studies analyse waste disposal methods from a macro perspective.

To enhance the recycling potentials and the entire waste management system, HEIs have the advantage of raising public awareness with respect to reduction, reuse and recycle (3R) strategies. Through proper and prompt planning, waste education and strict adherence to waste policies and procedure, 3R culture has been fostered more easily within the campus communities (Suwartha and Sari, 2013). Despite the multiple constraints and limitations of waste management in HEIs, promoting environmental education or related aspects in HEIs seems to be an imperative and unavoidable route to raise people’s awareness for long-term waste minimization. Also, the effectiveness of waste treatment strategies is different in developed and developing countries. Voluntary measures are not sufficient to promote pro-environmental behaviour towards waste in the context of developing countries (Dawodu et al., 2022b), usually strong legislation is required such as those developed within various regions of Europe. At the same time, waste management plans must ensure that the introduction of recycling facilities does not lead to excessive consumption of resources (Smyth and Booth, 2010). Moreover, there is still a global need for creating a transparent evaluation system for assessing the efficacy of recycling systems in campuses in the long-term. Hence, the infrastructure, options, approaches, and operations of campus waste disposal management, such as related policies, training & educations, public awareness need further observation, analysis, and enhancements.

#### 3.3.5 Building dimension (33 papers)

**Figure 10:**
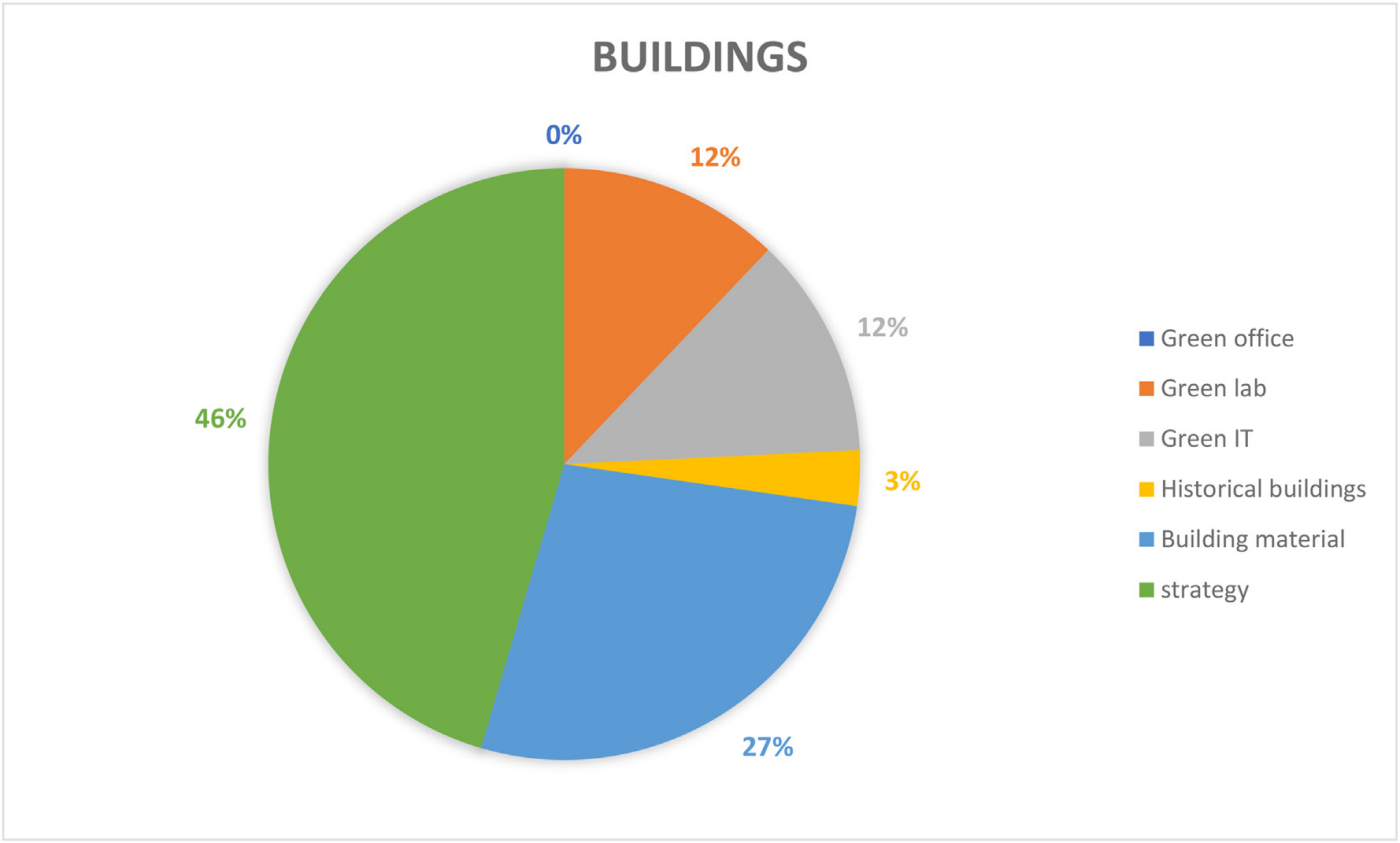
Coverage on the topic of building.

Buildings serve as the carriers of sustainable education. classrooms, laboratories, dormitories, and other types of rooms are largely used for campus purposes, for which strategies on architecture itself (46%) account for the largest proportion (see Figure 10). It is well known that teaching methods can affect the performance of students, but classroom organization is noted to affect the entire educational institution (Cebrián and Mogas, 2020). Indoor environments of classrooms depending on building location and its orientations can have significant impacts on its occupants when teaching activities are in process, this can be both physical and psychological. Typical factors may involve air pollution due to poor ventilation and other pollutants which can lead to instant and/or chronic health issues or risks. Also, closeness to a noise source with inefficient sound absorption materials and large empty space due to poor arrangement of chair and desk allocation can affect student’s and instructors’ health and wellbeing, their attention span and their teaching and learning performance. To reduce the cognitive burden of students and realize the purpose of meaningful and ontology construction among students, Cebrián and Mogas (2020) further argues that buildings may need to reconsider the physical and virtual learning spaces considering modern sustainable development targets by creating a more intelligent, personalized, and adaptive learning environments.

Green offices (0%) were not well accounted for because campuses generally possess traditional offices, which are more concerned with the disposal of internal related resources such as paper and equipment use, electricity use and management of staff and stakeholders. However green offices address the above-mentioned issues as well as tackling the environmental challenges present in the office. A case in point is the University of Malaysia's green office campaign which resulted in a reduction of 4,414,196.2 GJ/t of energy and CO_2_ emissions. This reduction was due to the ‘saving paper campaign under Green Office initiative’ (Zen et al., 2020). In fact, Un-printing is the biggest energy saved (95%) compared to other effort such as electronic-paper (85%), recycling (76%) and incineration (74%) (Counsell and Allwood, 2007). Thus, green office concept should be introduced to student and/or staff led start-up companies based on campus grounds to guide towards more sustainable building operation that enhance the perception, impact, and quality of their business. It is well known that buildings are one of the main contributors of energy consumption, therefore, targeting buildings for sustainable campus development can significantly reduce its energy consumption hence reduce its carbon footprints and emissions. Moreover, the contemporary corporate campus is not only a physical workplace for employers, but also a symbolic cultural and social environment that potentially promotes the ideology sustainability to students, clients and other stakeholders. Additionally, campuses are closely connected with the cities they reside in and become an important landmark of the city, thus emphasizing its importance in the urban environment (Bayas Aldaz et al., 2020). Yet, a major limitation is that green spaces such as offices are not as practical as they might seem due to its highly expensive initial costs, such that the energy to cost benefit may remain unconvincing to stakeholders. Li et al. (2014) stated that prejudice on the initial cost of green building is one of the key hurdles in green building development. It is also argued that benefits of green buildings are usually socially shared, while initial cost is borne by developers. As a result, this kind of imbalance between interests and cost undermines the promotion of green constructions. Hence, future attention is suggested to focus on facilitating the implementation of green buildings development in various scenarios that allows for direct benefits to the developer and office users as well as indirect benefits to others.

Smart technologies and intelligent robots that monitor soil condition, save transportation through flexible telepresence, monitor environmental conditions, provide hospitality service and even execute some tasks to improve recycling and sustainable manufacturing have slowly gained traction on the last half decade (Pujol and Tomás, 2020). Simultaneously, campus sustainability with smart building projects has also gained traction over the years. Ultimately, more smart machines and robots are expected to be utilized in construction and operations of buildings. With the growing advances in digital technologies and smart applications, Internet of Things (IoT), big data and artificial intelligence offers the basis for smart livings while controlling appliances and interconnected devices like surveillance cameras, access control, and heating/cooling systems. These operations have been widely used to provide “smartness” of buildings and infrastructures (Min-Allah and Alrashed, 2020). In a conceptual model of the universal smart campus proposed by Min-Allalh and Alreashed (2020), six pillars of smartness are highlighted including smart microgrid, smart utility, resource management, improved services, people management and educational services. Smart buildings are listed twice as associated aids for smart micro grid and people management respectively (Min-Allah and Alrashed, 2020). In another study on key performance indicators (KPIs) for smart campus and microgrid, both smart microgrid and smart buildings are listed as key service areas out of 15 service areas (Alrashed, 2020). Other aspects directly linked to campus sustainability on the KPI lists include financial sustainability, climate resilience, water resource management, pollution & waste management, propagation model and public participation. This implies the connections and interactions between smartness and sustainability of HEIs campuses. Because HEIs’ campuses imitate cities in many respects with its standard operating procedures, buildings, campuses are more suitable in adhering to smart city model (Min-Allah and Alrashed, 2020). Accordingly, similar assessment frameworks can also be extended to smart cities through a bottom-up approach.

#### 3.3.6 Transportation dimension (56 papers)

**Figure 11:**
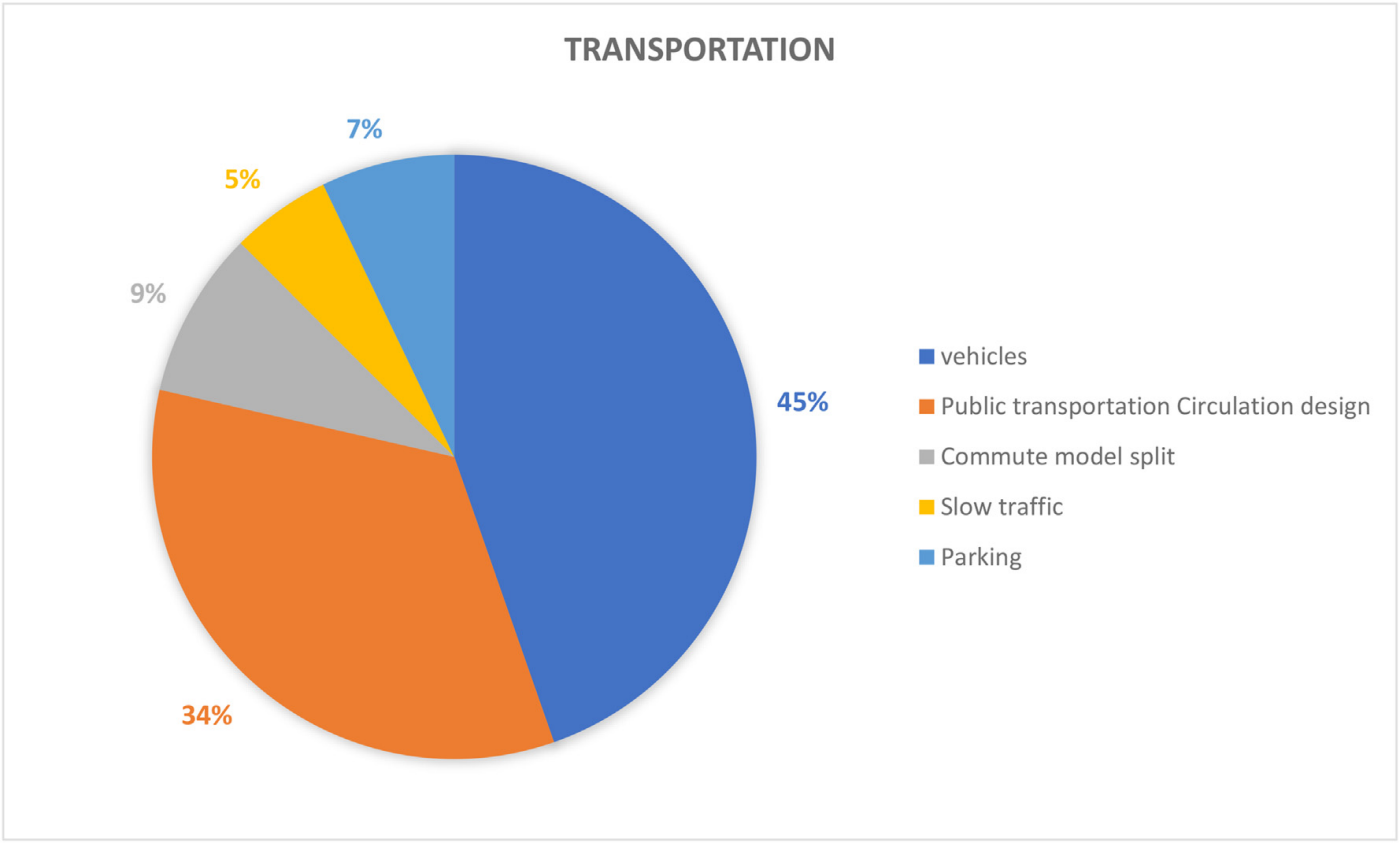
Coverage on the topic of transportation.

Most university institutions have a large carbon footprint, comparable to that of a small city, this study also shows that motor vehicles research accounts for 45% of transportation dimensions aligning clearly to the carbon footprint fact (see Figure 11). Vehicles here represents commuting of vehicles to campuses by various members of staff and students. The popularity of this topic is not surprising as significant emissions have been noted to plagues campuses. In fact, a comparative study showed the Qassim University emitted about 3.4MTCO_2_ (Net Carbon Footprint per person per year) accounting for 35% of campus emissions, which was then dwarfed by MIT which produced 36.4MTCO_2_ (Net Carbon Footprint per person per year) (Al-Mufadi et al., 2016). Also, transportation in HEIs involves not just all forms of transport but also what types of transport is predominant in campus DNA, generally classified under commute modal split or percentage contributions of a specific form of transport. This considers about 9% of campus research based on data. Ultimately such information, informs the development of sustainable campus strategies as wells the social, financial and environmental temperament of campus users. Generally, on-campus transportation has different sets of modes, including walking, skateboards, bikes, e-bike, electric walking tools etc. However, these tend to consider short commutes that range between 5 – 10 min. In fact, intra-campus travel still should ideally be shifted to electric or hybrid-based transportation, however such is not the case within numerous campuses around the globe (Norton et al., 2007). Páez and Whalen (2010) argue that universities around the world are more focused on the increasingly adverse effects of motor vehicles, and not the benefits and approach of alternative forms of transport. Though it seems this observation is more predominant within non-European cities. This element speaks to the need for optimizing public transport design and the infrastructure used to support them. Public transportation circulation design accounts 34% of the transport dimension, implying its importance in promoting campus transportation sustainability. While most campuses are designed for pedestrians, and more roads are pedestrian-friendly, students tend to be surrounded by the local culture of cars and schools are surrounded by communities that encourage driving (Kaplan, 2015). In fact, a key conundrum is the fact that universities actively seek more students to enrol each year and with population increase and need for specialized employment this means increased number of campus goers. This in turn results in campuses increasing the needs to develop parking lots and increase the size and number of lanes, which leads to further land use, high cost of parking lots, and reductions in the amount of green space on campus (McKenna and Altringer, 2021). This highlights the impact of external factors on campus sustainability decisions.

There is also a case for the lack of investigation on the use of public transportation within HEIs. For example, in China, public transportation studies within HEIs are quite limited and this is perplexing considering that most of Chinese HEIs’ campuses are quite big. With the large urban population and personal vehicles in China, the use of accessible public transportation design within campuses can be explored as a means of supporting daily commute to and from campus while reducing personal vehicle usages. For instance, it was gathered that 42 percent of college students drive to school alone in Virginia, contributing significantly to negative environmental, economic, and health impacts (Zhou, 2019). Therefore, policies related to reducing the use of personal vehicles can help campuses ensure the most efficient allocation of university land and funds, improve the quality of life, and reduce congestion on and off campus. Furthermore, external influences and shocks need to be investigated comprehensively to determine how best to maneuverer and mitigate negative influences of transportation that ripples across university sustainability strategies. Moreover, increased urban population has associated externalities such as high traffic which triggers a series of negative influences such as congestion, air and noise pollution and high car accident rate etc. To mitigate this, reducing traffic volume around the campus community has been the primary approach adopted by universities with added physical and mental health benefits to students (Kaplan, 2015).

In terms of traffic slowness (5%), small changes in the proportion of students walking or cycling can make a big difference in traffic patterns, hence making this less of a concern (McKenna and Altringer, 2021). Simultaneously, slow traffic can be alleviated with fewer cars. So, reducing the problems caused by excessive traffic and improving infrastructure will have a more positive impact on sustainability than the impact of slow traffic. Addressing traffic problems have various positive impacts including environmental and economic benefits, health & well-being benefits, improving public green awareness and strengthening cooperation with surrounding communities and businesses (Kaplan, 2015). Yet its obstacles and constraints are obvious. Firstly, undertaking constructions for transportation infrastructure upgrades can be cost intensive to HEIs (McKenna and Altringer, 2021). Secondly, it is very difficult to implement related policies as they need to be tailored to local conditions such as travel time, transportation costs, travel reliability, frequency of travel opportunities, and distance categories (Cattaneo et al., 2018). Many universities rely on Traffic Demand Management (TDM) tools that address traffic behaviour to discourage car travel and promote active mode sharing (e.g., cycling, walking, or using public transport) strategies (Rybarczyk and Gallagher, 2014). However, some studies have indicated that the analysis of campus transportation using TDM tools are not comprehensive enough to measure the effects of HEIs’ related programs.

To conclude, transportation within campuses much like waste management is key source of emissions controls and has an active role to play in the health and wellbeing of campuses. Universities should use every planning tool at their disposal to understand the geographical, environmental, economic and social characteristics of their campus to develop their own holistic plan that addresses modes of transport, transport technology, transportation design and infrastructure, traffic and the transport temperament of those on campus.

#### 3.3.7 Operations-social dimension (55 papers)

**Figure 12:**
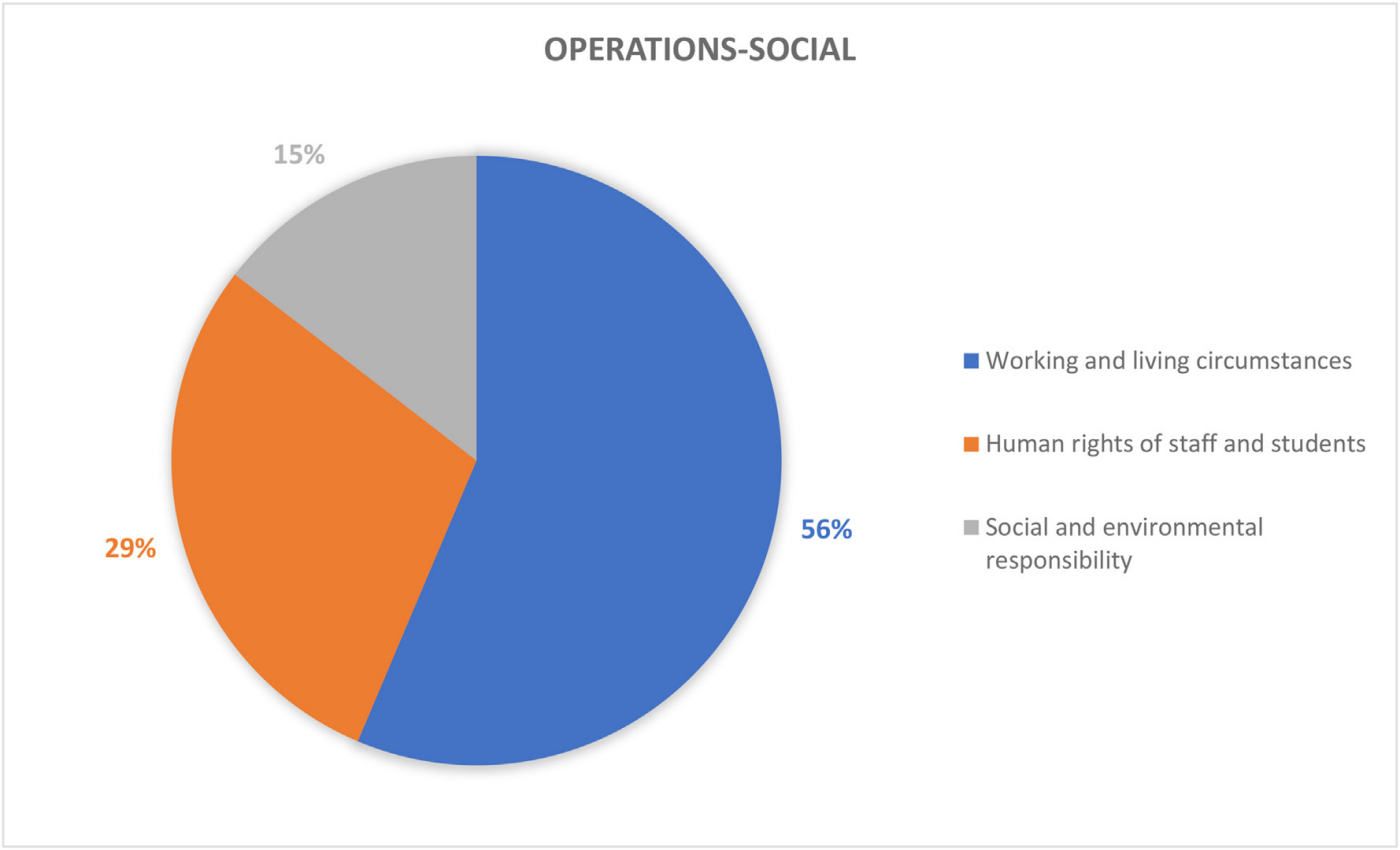
Coverage on the topic of operations-social.

Operations-social of HEIs refers to social and humanitarian rights of people on campus as well as their responsibilities. It also refers to the working and living circumstances and how comfortable people are within the campus environment. In Figure 12, the working and living environment accounts for the largest proportion of this dimension (56%), further demonstrating the influence of the environment on people's social life. Essentially, the interior environment of the building is quite important. The interior environment is usually related to the materials of the building, the facilities in the building and the use of the building space. As a result, conditions of indoor environments connect directly to social and environmental sustainability factors such as energy consumption, air quality, temperature, pollution, people comfortability, health, and wellbeing etc. The next three subsections expand on the three research observations in Figure 12. This is due to the broad nature of those elements.

##### 3.3.7.1 Working and living circumstances (31 papers)

**Figure 13:**
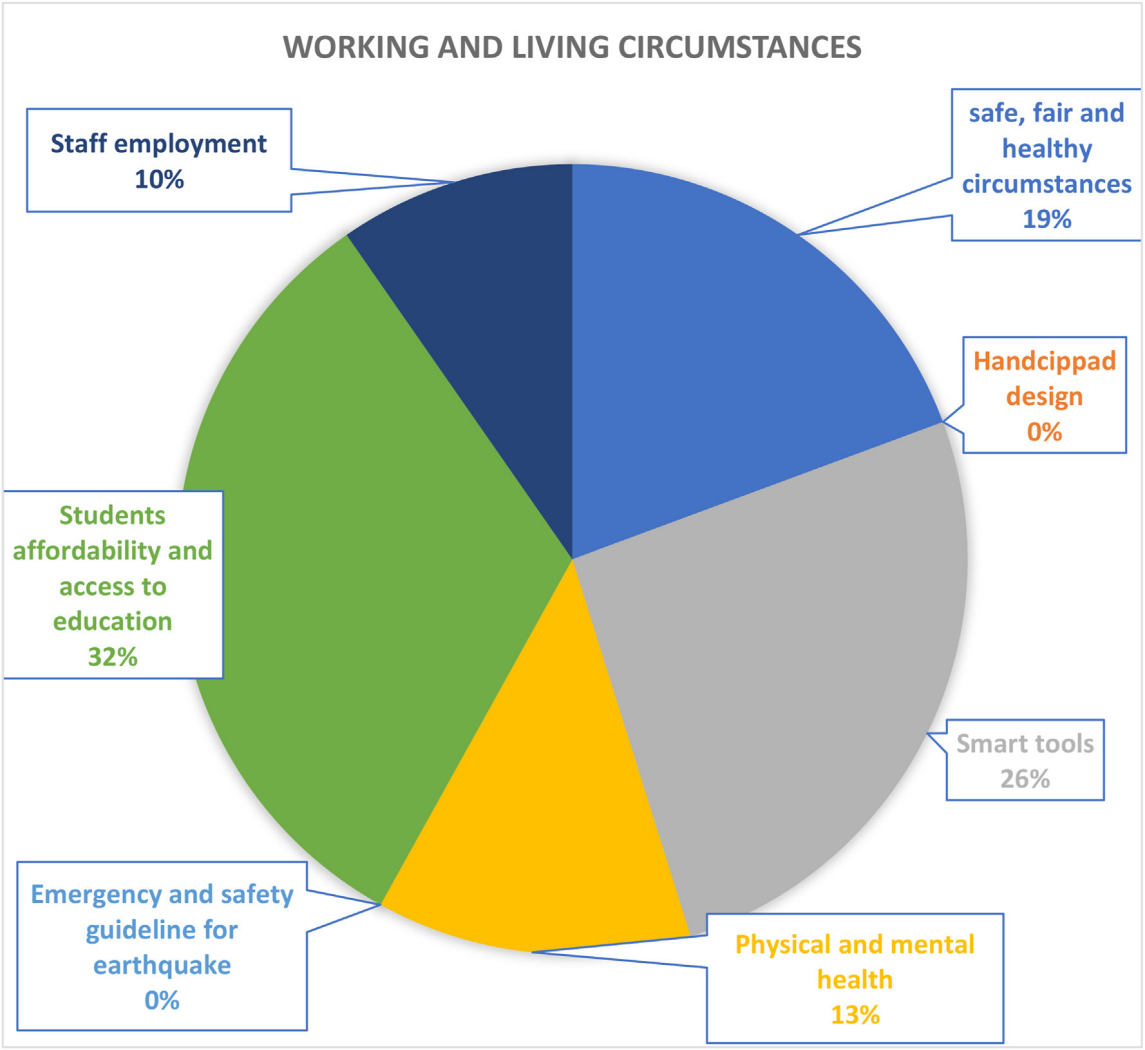
Coverage of working and living circumstances under operations-social dimension.

According to Figure 13, affordability, and access to education (32%) is the highest in the working and living circumstances. As education is the most important component of higher education institutions. Various types of novel learning methods are being researched and applied in education so that more students can conveniently acquire knowledge. On the other hand, smart tools (26%) and safe, equitable and healthy environments (19%) are key targets of higher education institutions in the modern era especially with the emergence of COVID-19. E-learning is an effective and sustainable teaching practice with a positive impact on students and environment. During the COVID-19 pandemic, it allowed for the continuation of teaching, learning activities. In a sense, E-learning has been viewed as a resiliency-based element of sustainability that has become an acceptable standard for living in the post COVID-19 era (Sales de Aguiar & Paterson, 2018). Till now, campus sustainable development research and tools tend to ignore the infrastructural aspect of resilience-based design and/or handicapped design. Evidence, as shown in Figure 13 indicate that 0% studies covered topics related to either the Handicapped Design or Emergency and Safety guideline for earthquake (Griffiths et al., 2019). This double down on the socially lacking and resilience-based issues of campus sustainability. Moreover, with people at the centre of this aspect of the dimension, their physical and mental health can be very effective indicators for determining whether their working and/or living circumstances are good or bad. This can be likened to the concepts of building sickness and sick building syndrome (Liu et al., 2020).

##### 3.3.7.2 Human rights of staff and students (16 papers)

**Figure 14:**
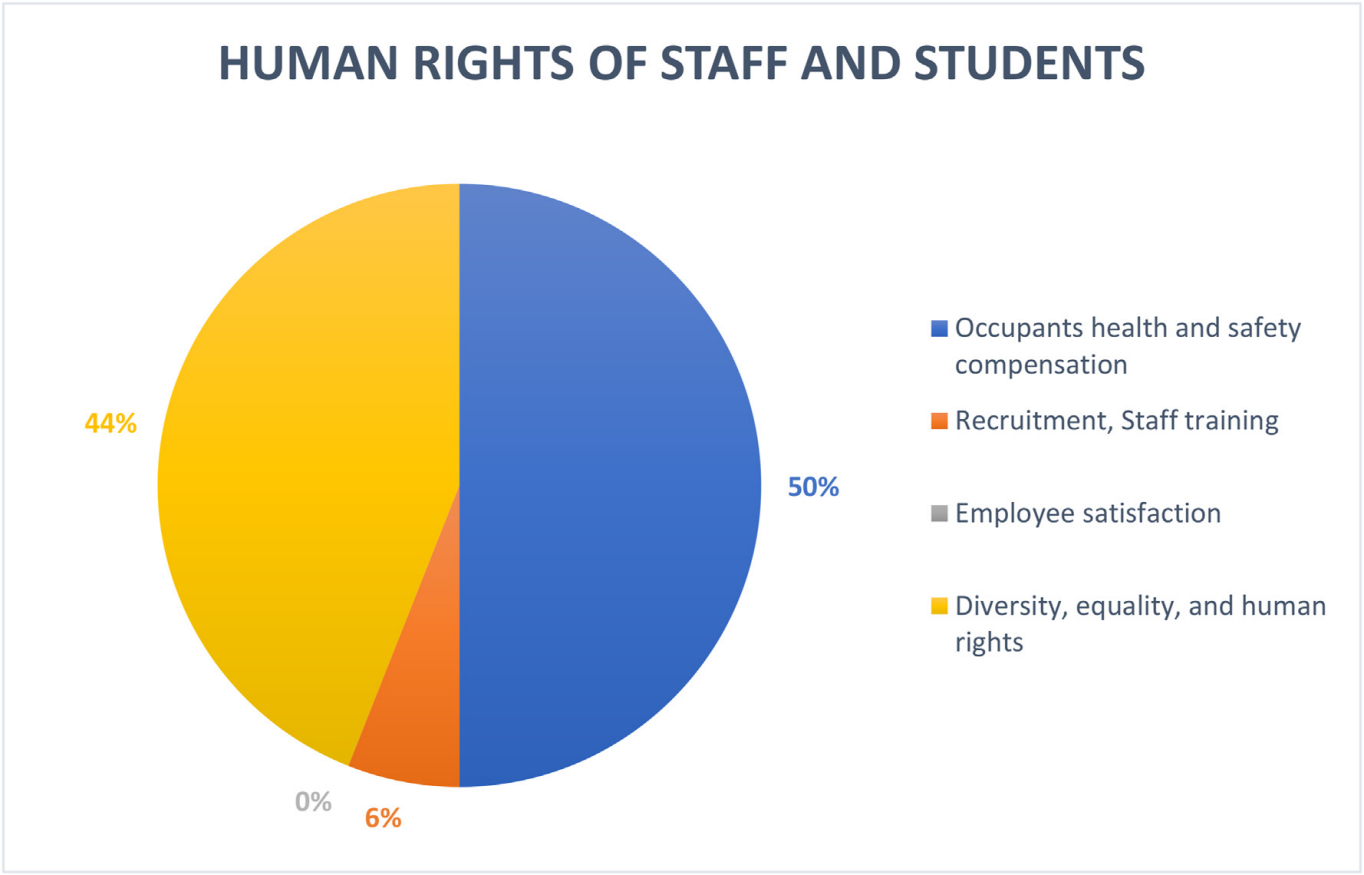
Coverage of human rights of staff and students under operations-social dimension.

Here, student and employee human rights, residents' health, and safety compensation (50%) were mostly considered, followed by diversity, equality, and human rights (44%). Meanwhile, recruitment/employee training (6%) were lower, with zero coverage of employee achievement (see Figure 14). This shows slight predisposition towards factors related to human rights. Considering the institutional stance of HEIs, the rights and responsibilities of personnel (employees) need to be paid attention, particularly in developing regions where such laws are lacking or are not often enforced. However, operational factors that affects functioning of HEIs and retaining of the community members such as employee training and developments require more effort.

##### 3.3.7.3 Social and environmental responsibility (8 papers)

**Figure 15:**
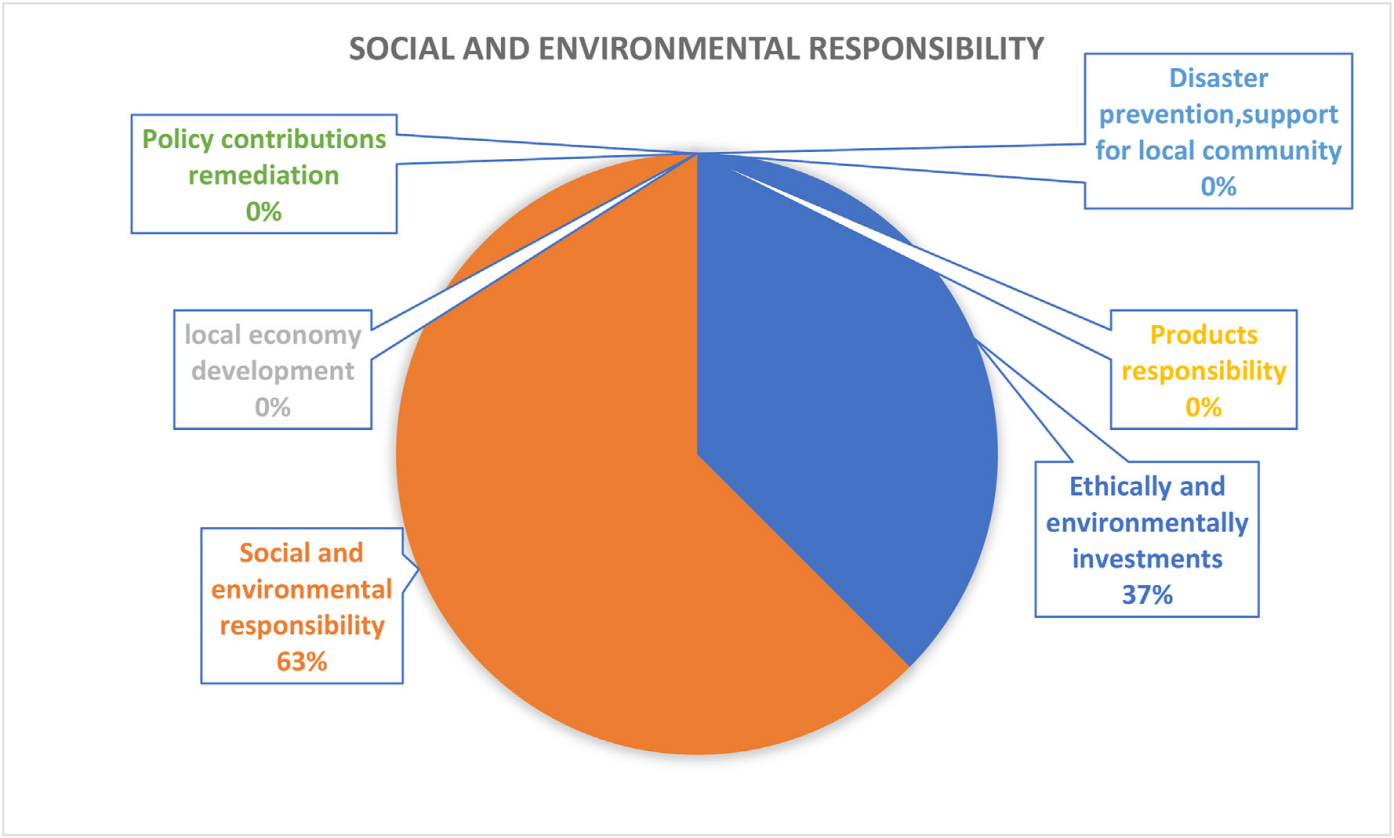
Coverage of social and environmental responsibility under operations-social dimension.

Figure 15 shows that social and environmental responsibility subsects contains the following six aspects: social and environmental responsibility; local economy development; policy contributions remediation; disaster, prevention, support for local community; products responsibility; and ethically and environmentally investments. However, the result of the literature review is occupied by only two parts, namely, social, and environmental responsibility (63%) and ethics and environmental investment (37%). Tiana and Villarreal (2016) claim that in the age of climate and pursuit of sustainability in education, teachers need to develop more inclusive, adaptive, and resilient ways of teaching. This was also due to the lack of sustainable patterns observed by teachers within Spanish infant and primary school education. Their argument advocated for practical activities through collaborative experiences that should be conducted in a real-life context. The aim was to enhance the output of sustainability and social responsibility program, which was in question. From a staff perspective, it is also argued that incorporating environmental responsibilities in a performance management system can make people aware and fully understand what they are expected to do as their environmental and social responsibility towards their campus (Anwar et al., 2020). As for people whose performance does not align with sustainability goals of an institution, Anwar et al. (2020) suggested that adopting disincentives as negative reinforcement can make people become more social and environmentally responsible towards the sustainability goal of that institution. In fact, observations revolving round enhancing social environmental responsibility show that not considering behavioural factors in environmental initiatives will lead to inefficient environmental performance. Thus, with the limited number of studies (8 papers) on the subject matter relative to campuses, there is great scope for further investigation, also evidence points to a need of a more balanced consideration of the other 4 dimensions not considered within the social and environmental responsibility dimension. It can be suggested that future research should delve into some of these overlooked aspects. This requires more comprehensive discussions from political, ethical, and economical perspectives.

#### 3.3.8 Operations-financial dimension (sustainability development investment) (32 papers)

**Figure 16:**
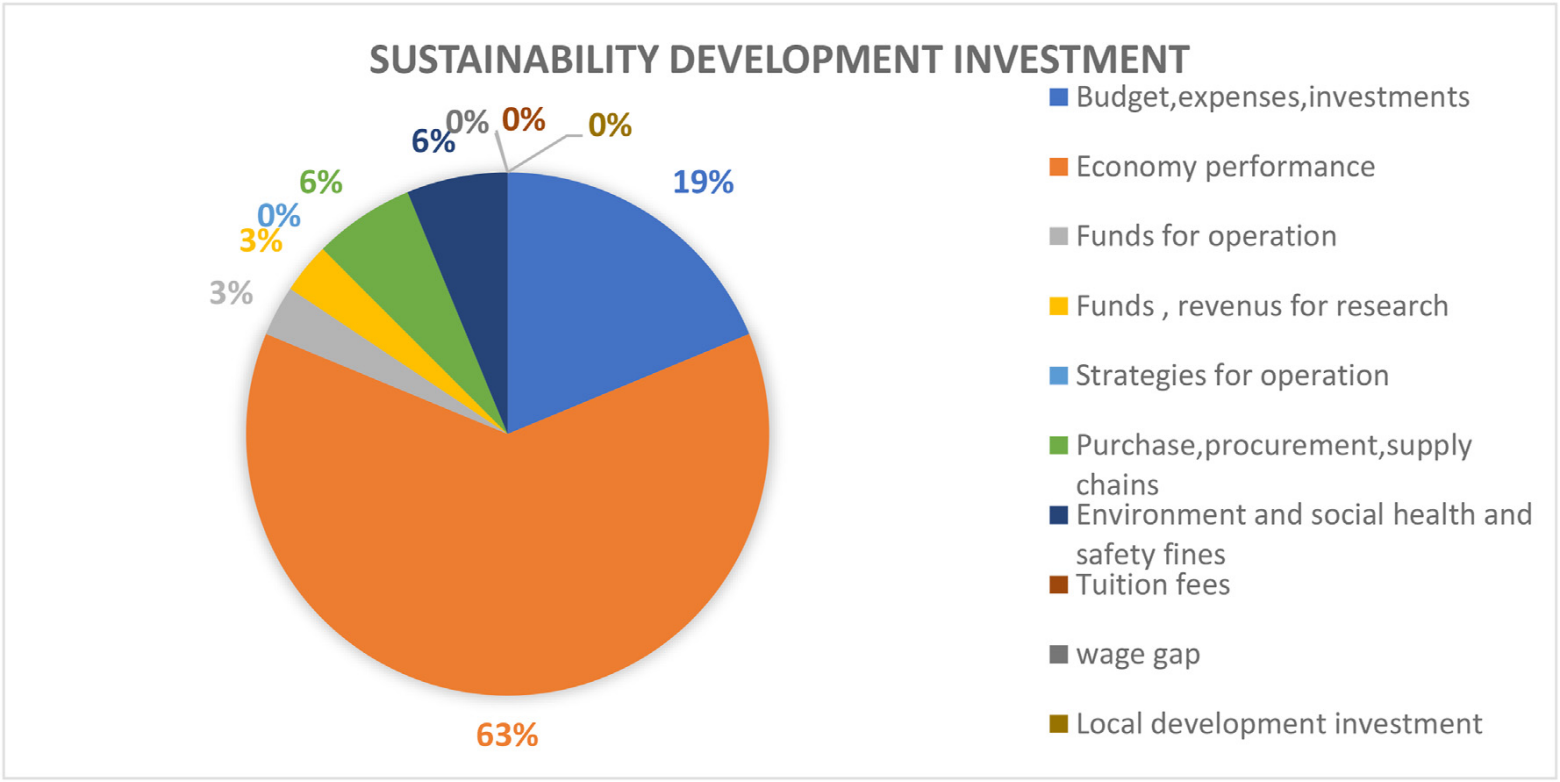
Coverage on the topic of sustainability development investment.

Sustainable development investment can be regarded as any financial investment for achieving sustainability goals. This topic covers a diverse aspect, making significant influences at multiple levels because most sustainable development projects require investments. For instance, green buildings, sustainable energy consumption, etc. Economy performance (63%) account for the largest factor in this dimension, implying that stakeholders consider economic performance as a primary indicators and determinants in the sustainability of campuses activities (see Figure 16). Further, a key agenda or concern for investors is the financial viability of investments in these technologies, and not just the environmental benefits. Meanwhile, most renewable energy equipment requires large initial capital and follow-up maintenance costs. Additionally, research into technological innovation of renewable energy needs significant funding. For example, a passive rainwater harvesting system was used in the Roof of the building of Engineering Faculty of Yalova University, which is expected to save €2,900 per year and 8.5 tons of water per year (ERBIYIK et al., 2021). The National Autonomous University of Mexico implemented a solar photovoltaic system, which went on to save about $12,089 per year and associated green greenhouse gas emissions (Hernandez-Escobedo et al., 2020). Texas State University (TSU) saves its campus 15,391,436 kW-hours of electricity per year (17% of its annual energy costs) with sustainable energy installations. This includes over $1,000,000 in annual cost savings, with the minimum payback period of nearly a year for the installation of smart pumps. Lighting replacement has also had the most significant annual savings of $5,201,804. Solar panel installation had the highest upfront investment but avoided 2,926.81 metric tons of carbon dioxide per year (Hashim et al., 2020). In sum, sustainable investment is more prone to address the three dimensions of sustainability holistically with its direct focus on at least the environmental and social aspects of sustainability (Dawodu et al., 2017).

According to Figure 16, Tuition fee (0%), wage gap (0%), local development investment (0%) and operation strategy (0%) are not covered, all of which focus on person-to-person transactions. Today, many large public HEIs are facing rising demand for space and energy as their student population grows rapidly. According to Hashim et al. (2020), it is important for universities to adopt feasible energy efficiency investment plans that range from procurement and supply to installation and operation. These measures can help grow the sustainability of any campus but in many cases, they can be costly and difficult to implement and require increased participation, action, and awareness by campus residents. Accordingly, replacing inefficient lighting with an energy efficient option seems like an easy strategy, but when educating students and staff on turning off unused equipment remains a challenge. Such behavioural issues can hinder sustainable development investments.

#### 3.3.9 Education dimension (162 papers)

**Figure 17:**
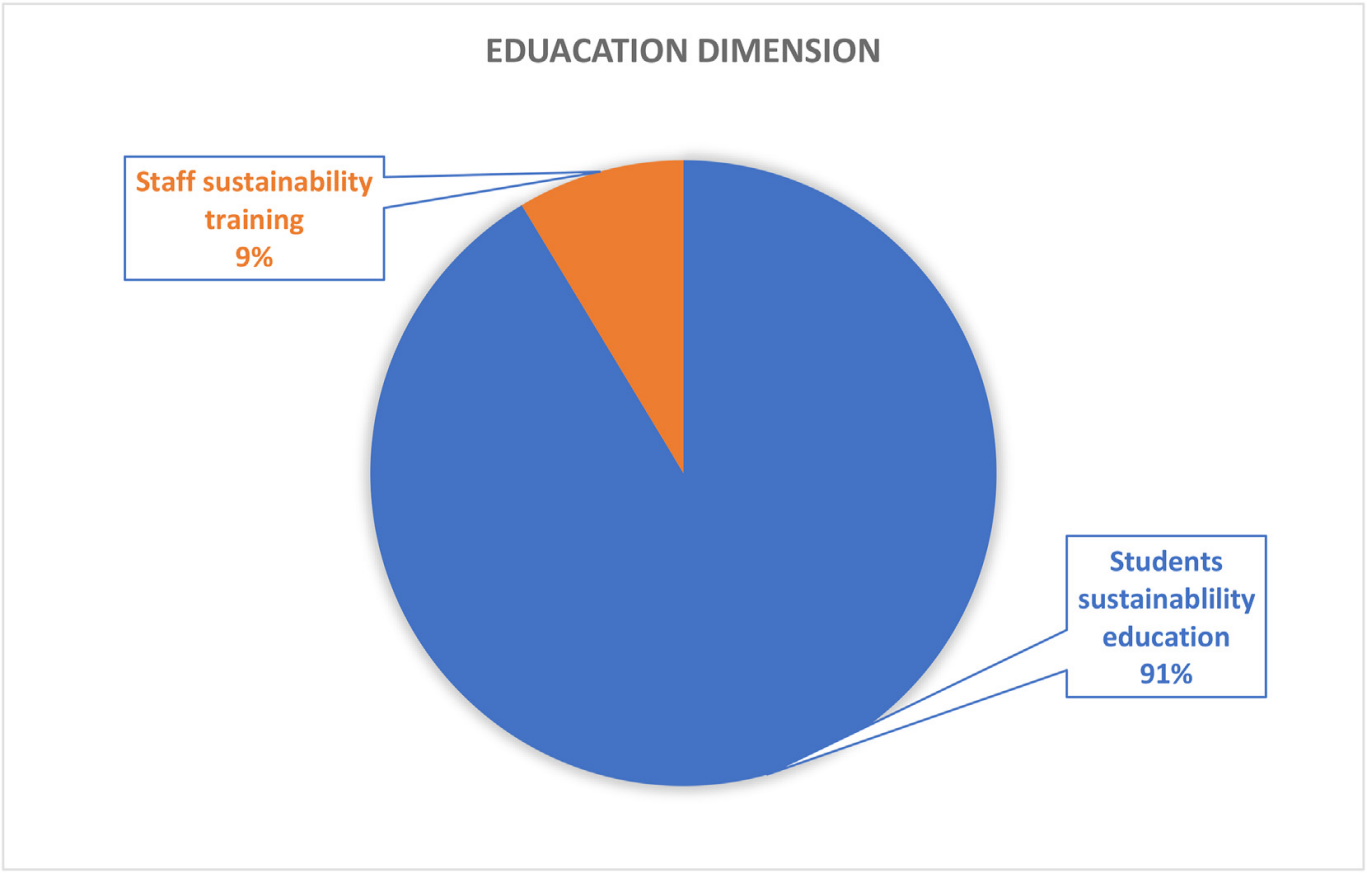
Coverage on the topic of education dimension.

Education as the main function of HEIs is extremely important for sustainable development. In developing countries like China, people's awareness of sustainable development is relatively weak, so education is an important way to improve students' awareness. Figure 17 shows that Sustainable education for students (91%) accounts for a large proportion of this dimension. As change agents and leaders in the future of sustainable development, it is expected that more papers should be devoted to students’ education on sustainable development. Compared to students’ sustainable education, the proportion of staff sustainable training is significantly lower (9%). The underlying reason for this phenomenon could be that employees are the way through which sustainable education is introduced to students, so more studies are conducted to explore the methods of student education rather than employee-oriented research. Secondly, the population ratio of students over staffs might also influence on research coverage. Since the majority of residents are students. Thirdly, assumptions that university staffs already have sustainability knowledge, or they could learn by themselves due to their high level of intelligence and professional skills. Lastly, it might be difficult for HEIs to find suitable personnel and/or approach to lecture their staffs on the topic of campus sustainability. Overall, sustainability training and its impact on staff is still an emerging area of research and has only been recently studied.

##### 3.3.9.1 Students sustainability education (148 papers)

**Figure 18:**
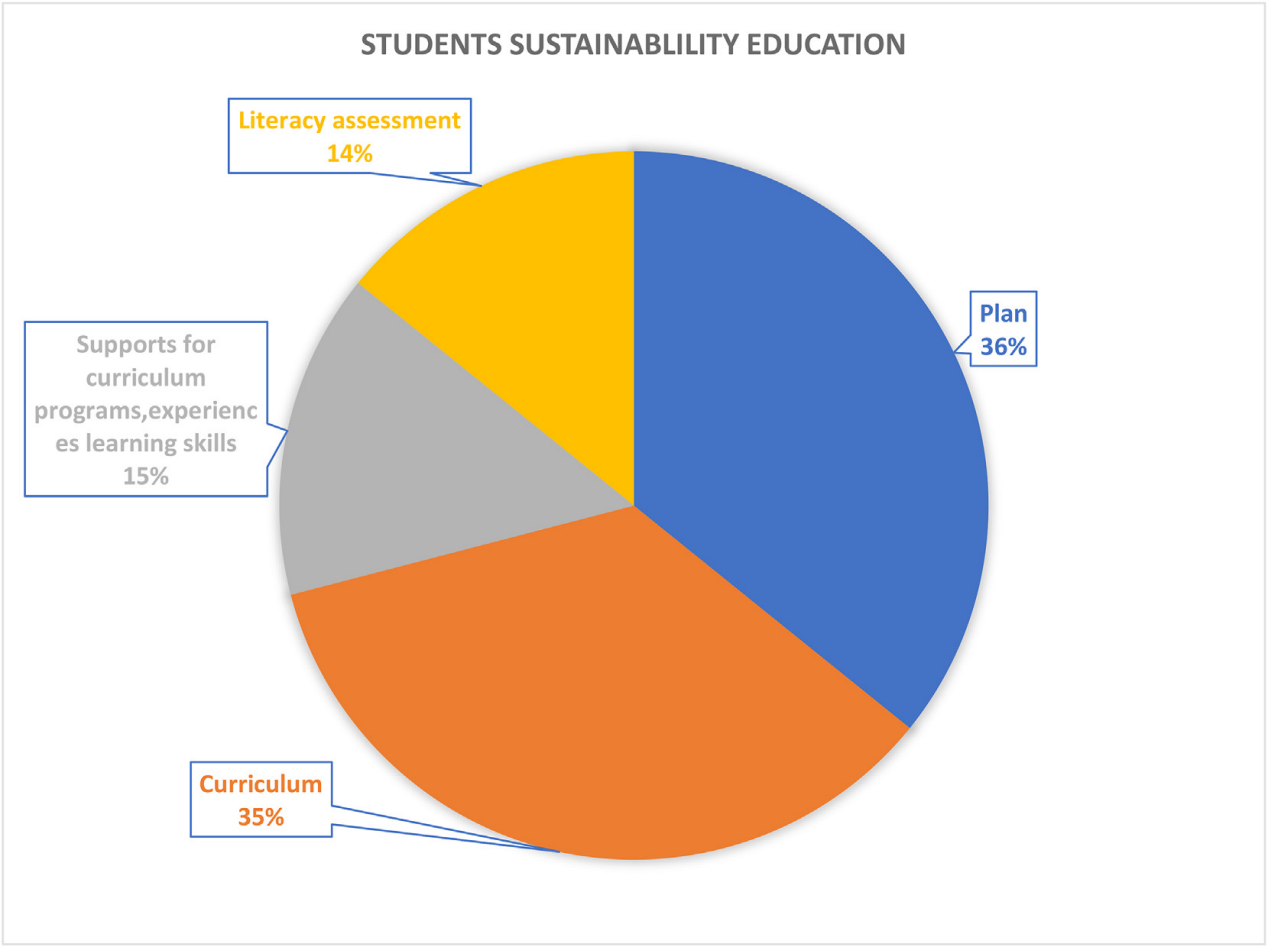
Coverage on the topic of students’ sustainability education.

In Figure 18, Plans (36%) and courses (35%) make up the largest proportion of sustainable education for students, while supporting lesson plans, experiential learning skills (15%) and literacy assessment make up a smaller proportion. For the plan topic, it covers the overall planning of the school, such as the curriculum, the organization of activities, and the future strategic development plan of campus sustainability in several years. It’s mainly about dealing with questions like whether the school has relevant courses; whether there are relevant teaching activities for sustainability; whether there will be any activities about sustainability in the future; and whether the school has any new curriculum or department plans for sustainability. Not only are plans and courses key to students' learning about sustainable development, but the knowledge in sustainable courses can be extended to other areas of expertise. The reason is that sustainability courses tend to be interdisciplinary in nature. For example, chemical engineer working sustainable jet fuel can end up as a policy maker or manager at aircraft industry geared towards sustainable fuel management. It is based on this that most courses within science, engineering, business and even humanities have elements of sustainable studies fused into them. This could range from abstract contents such as general knowledge on climate change to more specific and technical modules, which are mostly elective modules chosen by students based on their career or knowledge interest (Oxenswärdh and Persson-Fischier, 2020). Developing sustainability plan as part of course development is still in its formative years but progress towards this agenda is being made. For example, an assessment of sustainable website development and interaction was conducted on 96 universities in Canada, which helped thr universities improve the effectiveness of their sustainability plans (Amey et al., 2020). There are also digital media systems to help make learning sustainable. For example, e-learning systems have become not only a very important part of teaching, but also an aid to face-to-face learning, providing additional learning support for learners at school (Alharthi et al., 2019). It should be noted that a common mistake in academia is the explicit focus on the theoretical, often limiting the impact of the practical. The consequences of this becomes the lack of practical learning experience and skills. Current sustainability education is at an early stage and is currently focused on promoting sustainability awareness instead of implementing it effectively. Sustainability plans and any educational plan generally should actively involve the research informed and practice-based learning. An example of such infusion is service learning, where students learn in service of an organization with specific learning objectives and outcomes required (Bugallo-Rodríguez and Vega-Marcote, 2020). Furthermore, a shift away from macro-based policies of sustainable plans and education and a move towards well tailored development to improve students' individual sustainability is required.

##### 3.3.9.2 Staff sustainability training (14 papers)

**Figure 19:**
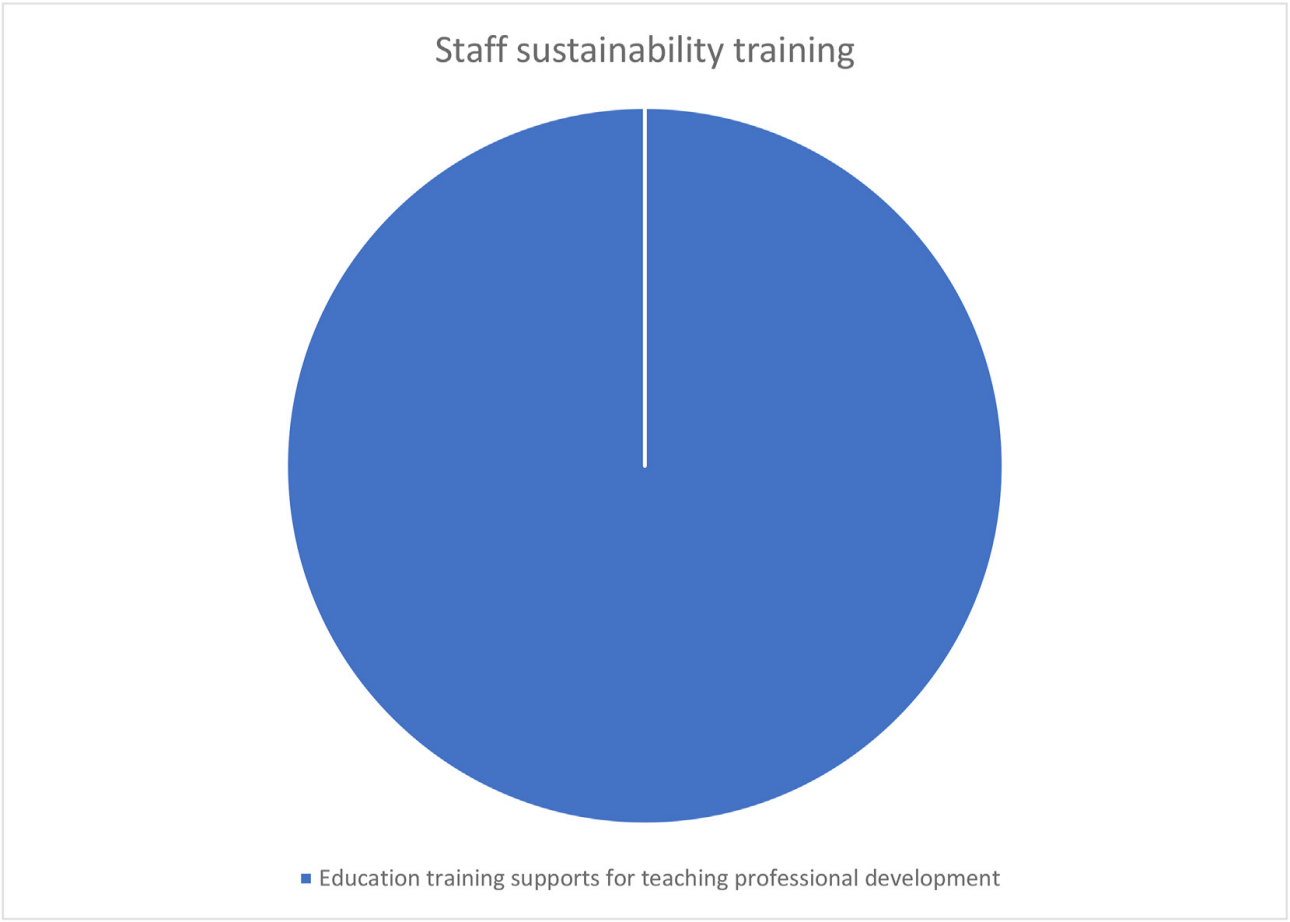
Coverage on the topic of staff sustainability training.

Regarding sustainability staff training in Figure 19, the only consideration appears in the literature is “education training supports for teaching professional development”. Some authors suggest integrating sustainability into the curriculum of teaching professional development with respect to specific context of each HEIs (Bugallo-Rodríguez and Vega-Marcote, 2020). This also implies the importance of context-specific strategies in campus sustainability development. Such training is already executed within the Postgraduate Certification for Higher Education (PGCHE) program and frameworks within the United Kingdom. However, such frameworks are generic and focus widely on academic teaching. More context specific training course on sustainable education is required, especially professional development courses that enhances not only knowledge on the sustainable agendas but also sustainable strategies and evaluation processes. Moreover, as with ‘student sustainable education’ supporting a learning approach based on practice and experiential activities are also recommended for educational training (Bugallo-Rodríguez and Vega-Marcote, 2020). The result of this subsection substantiates the assumptions of the possible reasons for lack of staff sustainability training in Section 3.3.9, revealing that both research and practices in the areas of campus sustainability education are still lacking with limited considerations and coverages of related topics. For staff sustainability training, future directions may involve development of a systematic training procedure or standards, inventing some certifications of training qualification, selection of training modes and contents, and clarifying HEI and staffs’ roles and responsibilities.

#### 3.3.10 Research dimension topic (69 papers)

**Figure 20:**
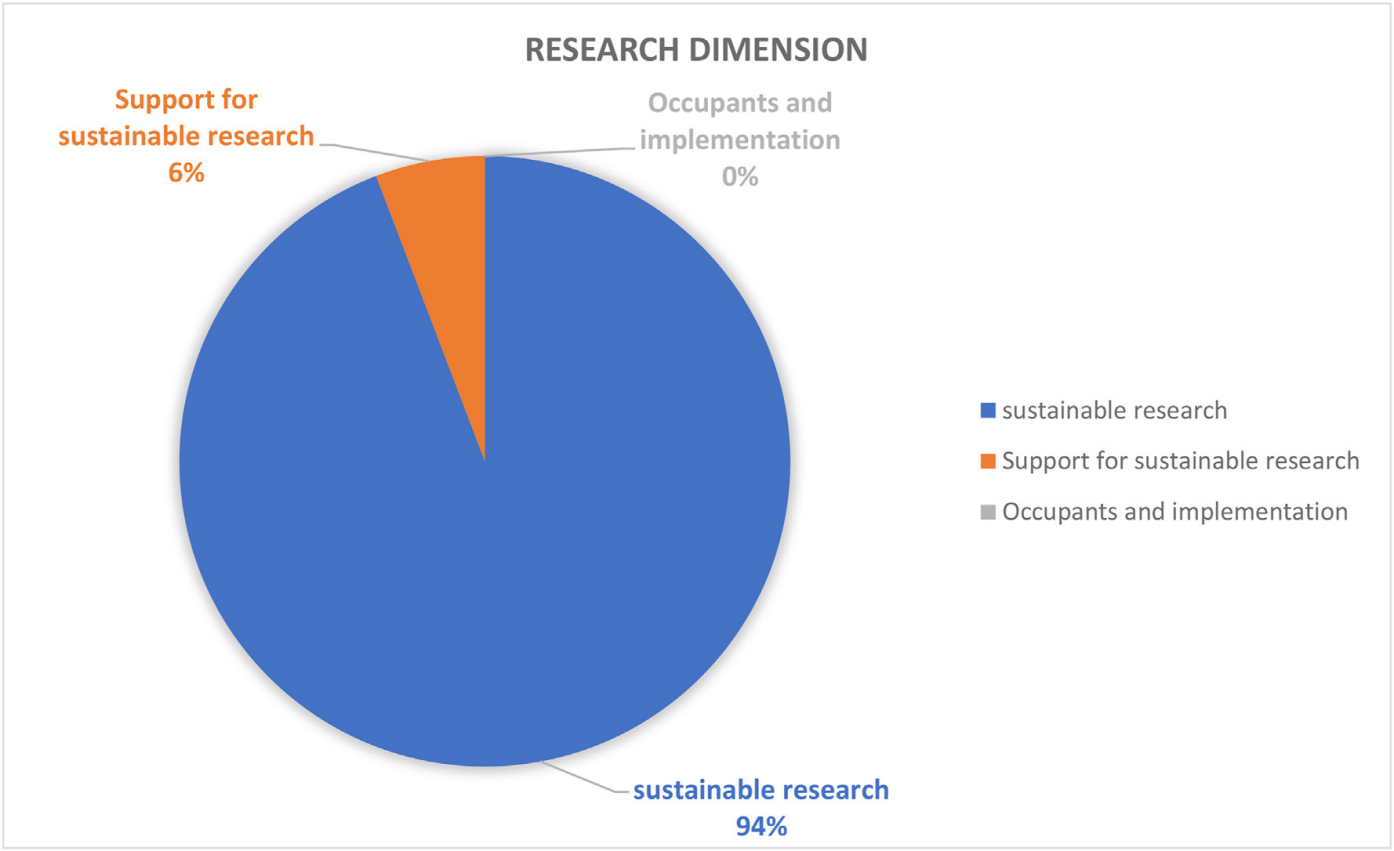
Coverage on the topic of research dimension.

In the 69 papers shown in Figure 20, Sustainability research covers the bulk of papers at 94%. These research focus on various investigation of sustainability or the pursuit of sustainability in universities and campuses, most of them focus on sustainable policies, as well as topics sustainable energy, economy, and technology. Studies in Figure 20 also includes various green evaluation systems similar to some CSATs such LEED, WELL, Green Metric, etc. The sustainable policies mentioned are mainly implemented at a building level, while other research focuses on the evaluation of sustainability from students’ perspective ranging from sustainable curriculum and experiences to the impact of certain clean technologies to campus or on student life. The support of sustainable research is also key topic, this occurring 6% of the time. Globally, the word ‘sustainable’ has attracted attention and numerous funding, such that most universities have an element of internal funding allocated to sustainability-based initiatives. Take the University of Nottingham China which offers over $10,000 on its campus change initiatives centred on sustainable solutions. Hence this indicator or agenda is a key metric to measure the value of sustainability either in the development of a campus tool or as a standalone initiative.

##### 3.3.10.1 Sustainable research (65 papers)

**Figure 21:**
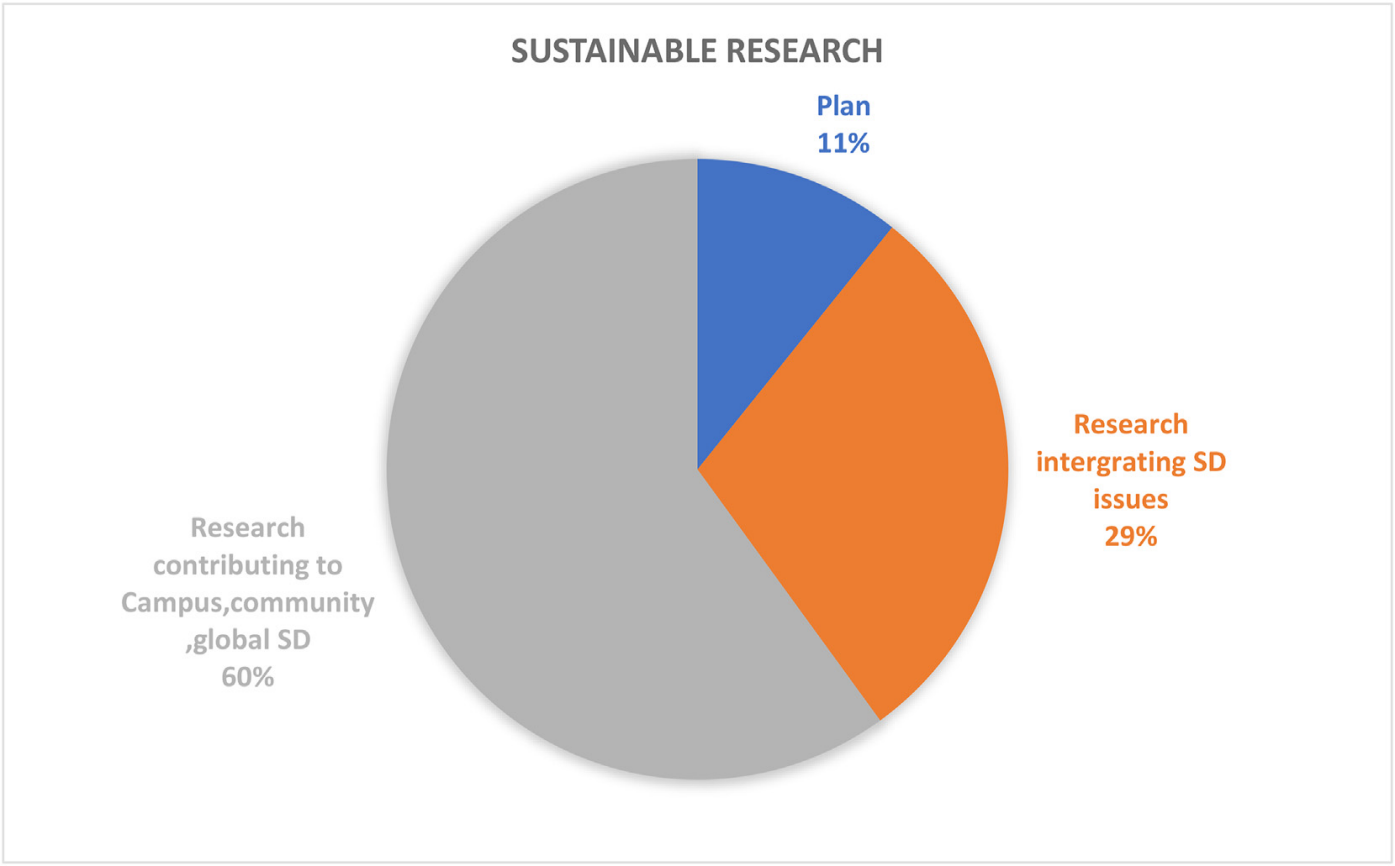
Coverage on the topic of sustainable research.

The highest proportion of sustainability studies (60%) are those that contribute to campus, national, and global sustainability (see Figure 21). All these studies have one thing in common: they focus on what is common on most campuses. In other words, these findings can be applied to almost all universities (Mawonde and Togo, 2019). The main advantage of sustainable research is that some of the results can be used as a guide, making it possible for HEIs to learn from others’ experiences. But this is also constrained by the differences among HEIs’ campuses, as the context differences may make successful sustainability strategies of one campus ineffective in another. For instance, campuses in areas of lower daily sunlight averages cannot directly imitate sustainable energy strategies used in area’s of higher daily sunlight averages. For example, such deviations determine if solar technologies is a viable option within two different campuses, further still such deviations determine the type of solar technology that may be more feasible in one location versus another. Moreover, it can also be argued that sustainability is transient phenomenon subject to time. For example, students' as well as other stakeholders on campus change annually, these social and institutional changes may have significant impact on the characteristics of the university thus changing the needs and wants of the campus. Such variations are generally not accounted for in currently existing CSATs, which generally take blanket approach to sustainability issues.

##### 3.3.10.2 Support for sustainable research (4 papers)

**Figure 22:**
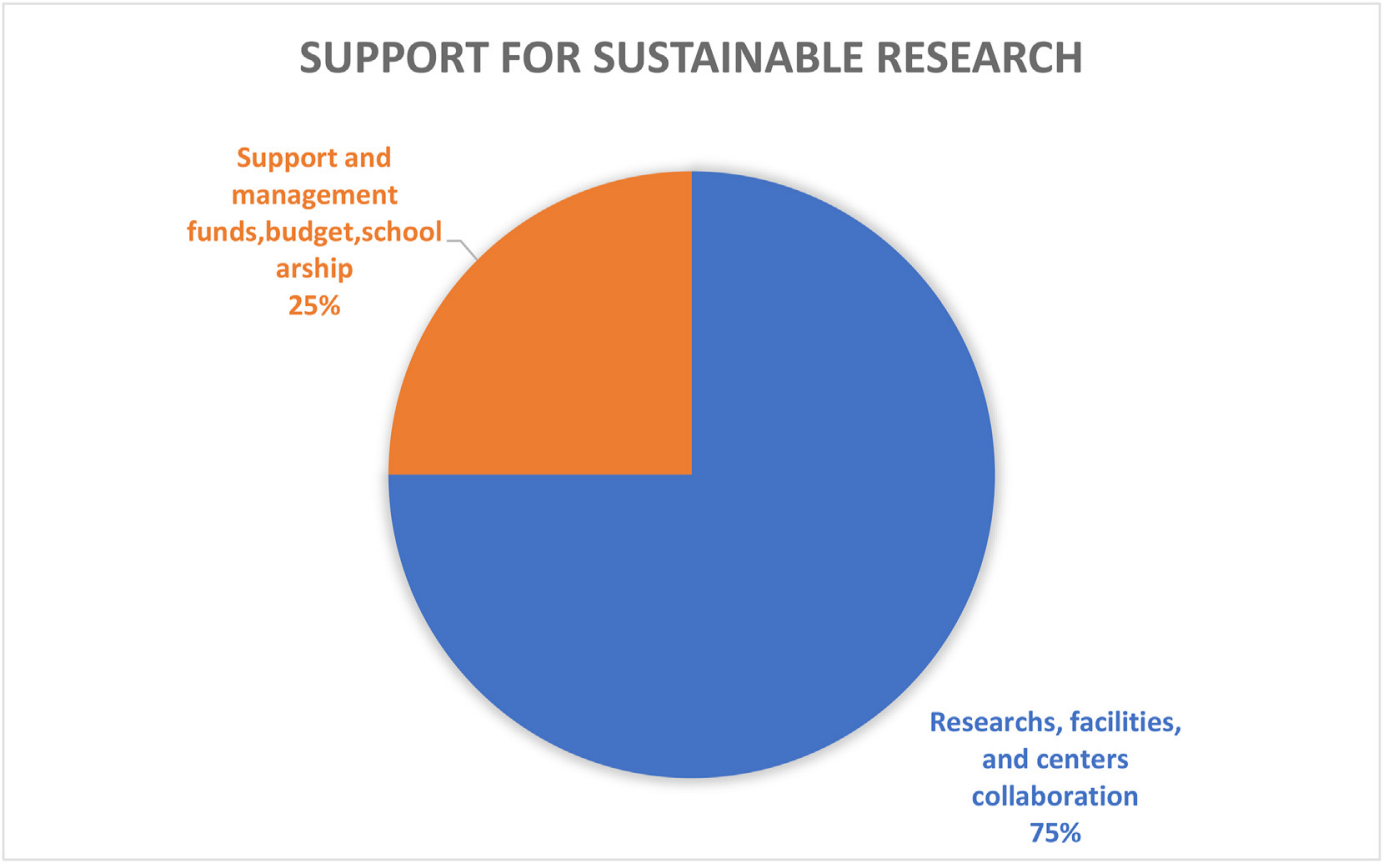
Coverage on the topic of support for sustainable research.

Support for sustainable development in Figure 22 represents 6% of the research dimension. Research, facilities, and centres account for 75%. Interestingly, the results of the study by Shealy (2016) suggest that simply holding a preliminary design meeting in a building that contains encouraging sustainability design features may encourage design teams to achieve the same high level of sustainability performance. Support, management, budget, and scholarship (25%) are considered less in sustainability research. This maybe because for most circumstances, this aspect has some overlaps with other aspects of other topics. For instance, budget and scholarship for sustainable research might be covered in those aspects of operations-financial topics like “*budget, expenses, inves*t*ment*” and/or “*funds, revenues for research*”. Scholarships that aid the less fortunate and provide increased diversity, inclusivity, and even resiliency is a key part of Education for Sustainable Development (ESD). Thus, finances aimed at supporting the disadvantaged is key to promoting ESD. However, the exact amount of what should be allocated requires further investigation. The benefits of supporting sustainability research is that it provides additional supports to sustainability development and offers new perspectives and support for various stakeholders from different demographics and background. Meanwhile, a major con is that some research results cannot always be translated directly into practices due to specific contexts and unique characteristics of different HEIs. Furthermore, promoting campus sustainability and their associated research is still limited in developing countries, in spite of the awareness being raised.

#### 3.3.11 Engagement-campus dimension (20 papers)

**Figure 23:**
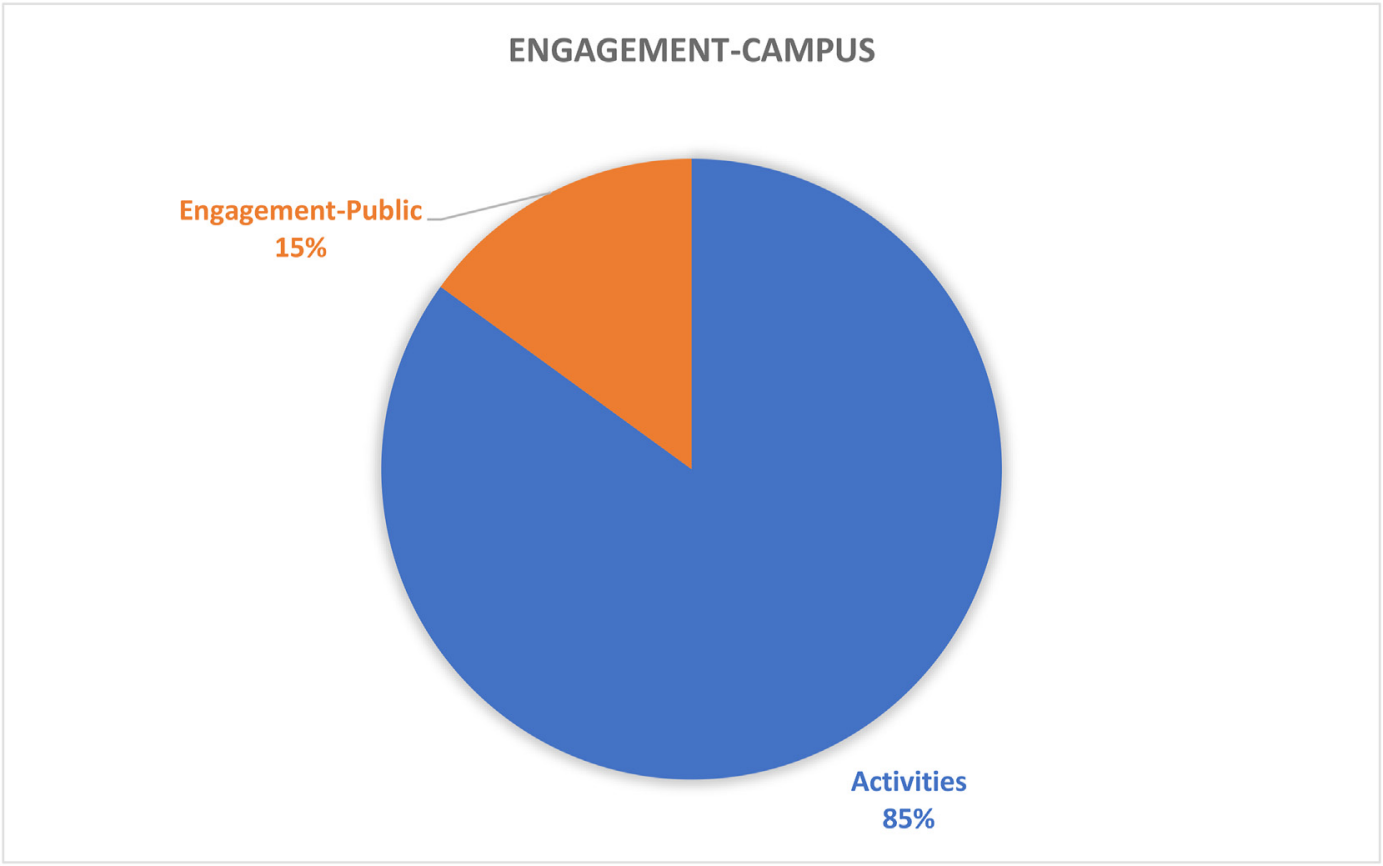
Coverage on the topic of engagement-campus.

The result in Figure 23 shows that the most popular approach of engagement on campuses is conducting activities (85%) while only a small proportion involve public engagement (15%). It is believed that engagement via campus-based project bring multiple benefits such as enhanced student experiences in contributing towards on-campus sustainability goals. A key approach to campus sustainability is enabling students to be better prepared to work in teams towards comprehensive goals; contributions to improving their research and management skills and furthering future goals in their professional careers, etc (Clark and Capps, 2020). Additionally, public engagement activities can be used as a very powerful propaganda tool for HEIs as well as an interactive way of fulfilling their social responsibility. It can improve HEIs’ reputation and public favourability through increasing exposure rate and strengthening connections and cooperation between HEIs and the public. Also, undertaking large-scale public engagement projects and activities will also make contributions to the communities’ sustainability goals, and even regional sustainability. If some engagement projects and activities can be replicated and applied to other HEIs, this will bring more co-benefits and may set them as flagship projects or benchmarks for campus sustainability engagement.

##### 3.3.11.1 Activities (17 papers)

**Figure 24:**
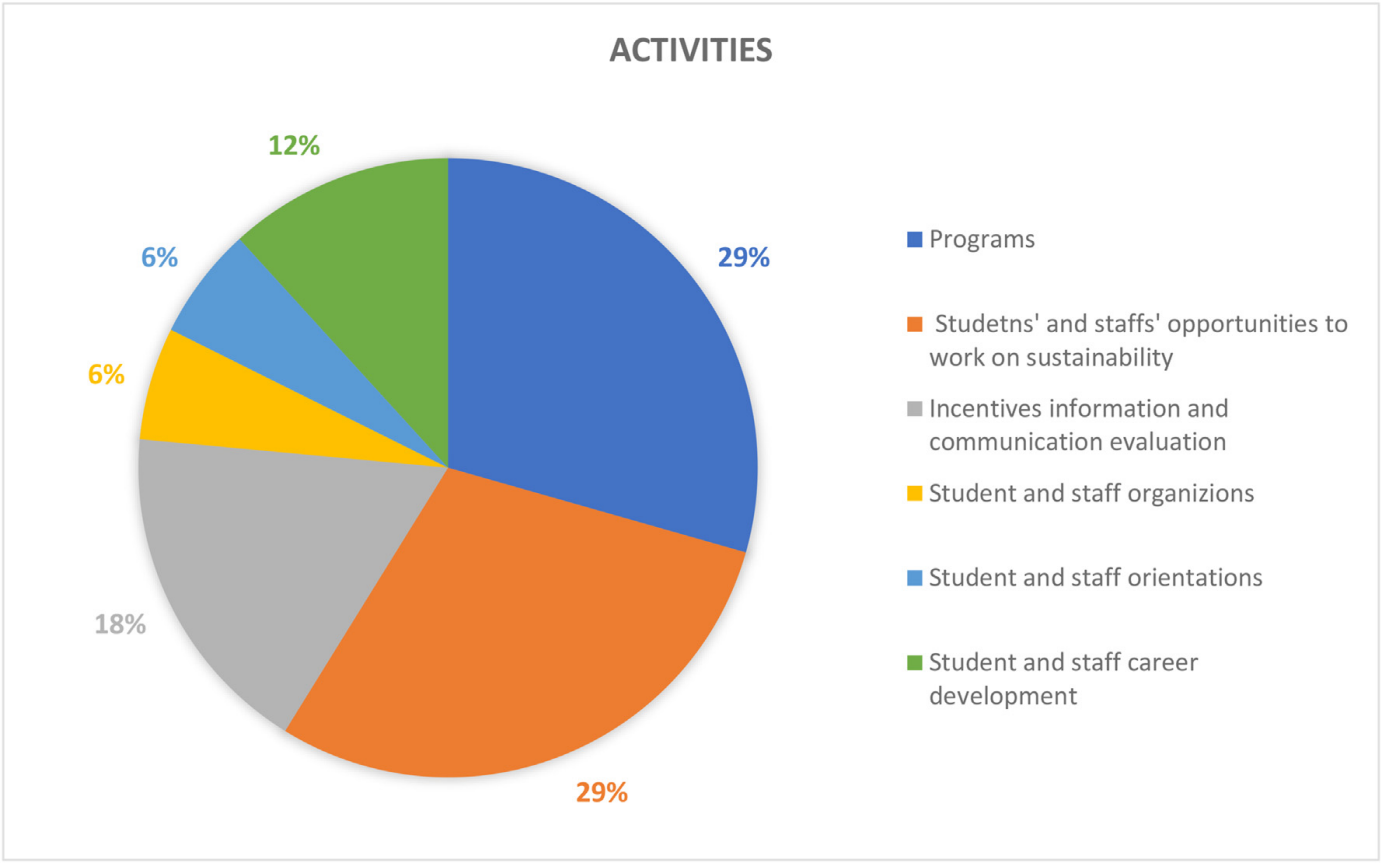
Coverage on the topic of activities.

There are 6 aspects that have been covered in the “activities” topic including programs, opportunities, incentive information, organizations, orientations, career development. Programs (29%) and opportunities for students and staff to engage in sustainable work (29%) accounted for most of the research papers (see Figure 24). The possible reason is that many data sources of sustainable frameworks and models come from students and need to be analysed through their behaviour patterns. This may be because sustainability programs are the fundamental basis for students and staffs to join campus engagement exercise, while the opportunities can be considered as a direct indicator for illustrating how easy and convenient it is to participate in those sustainability development programs. The literature suggests that engagement activities like interactive programs can promote sustainability awareness while increasing students and staff’s related knowledge. In a study on recycling entrepreneurial initiatives programs, the authors discover that those organizing the programs not only enhance sustainability knowledge and awareness but also encourage innovation and creative thinking in reusing recycled materials (Er et al., 2019). Besides that, Er et al. (2019) also pointed out other additional benefits including financial incomes, environmental protection and conservation, and campus environment enhancement. Essentially, engagement activities and programs can provide educational functions, indicating the great potential of integrating campus sustainability education and engagement activities in HEIs for co-benefits and to achieve campus sustainability goals more easily. Plus, related documents, outputs, and feedbacks of those programs can also be used in research for designing future programs, examining existing strategies, improving related policies, and even forming a structured feedback process for setting up an adaptive management system of campus sustainability in HEIs.

##### 3.3.11.2 Engagement-public (3 papers)

**Figure 25:**
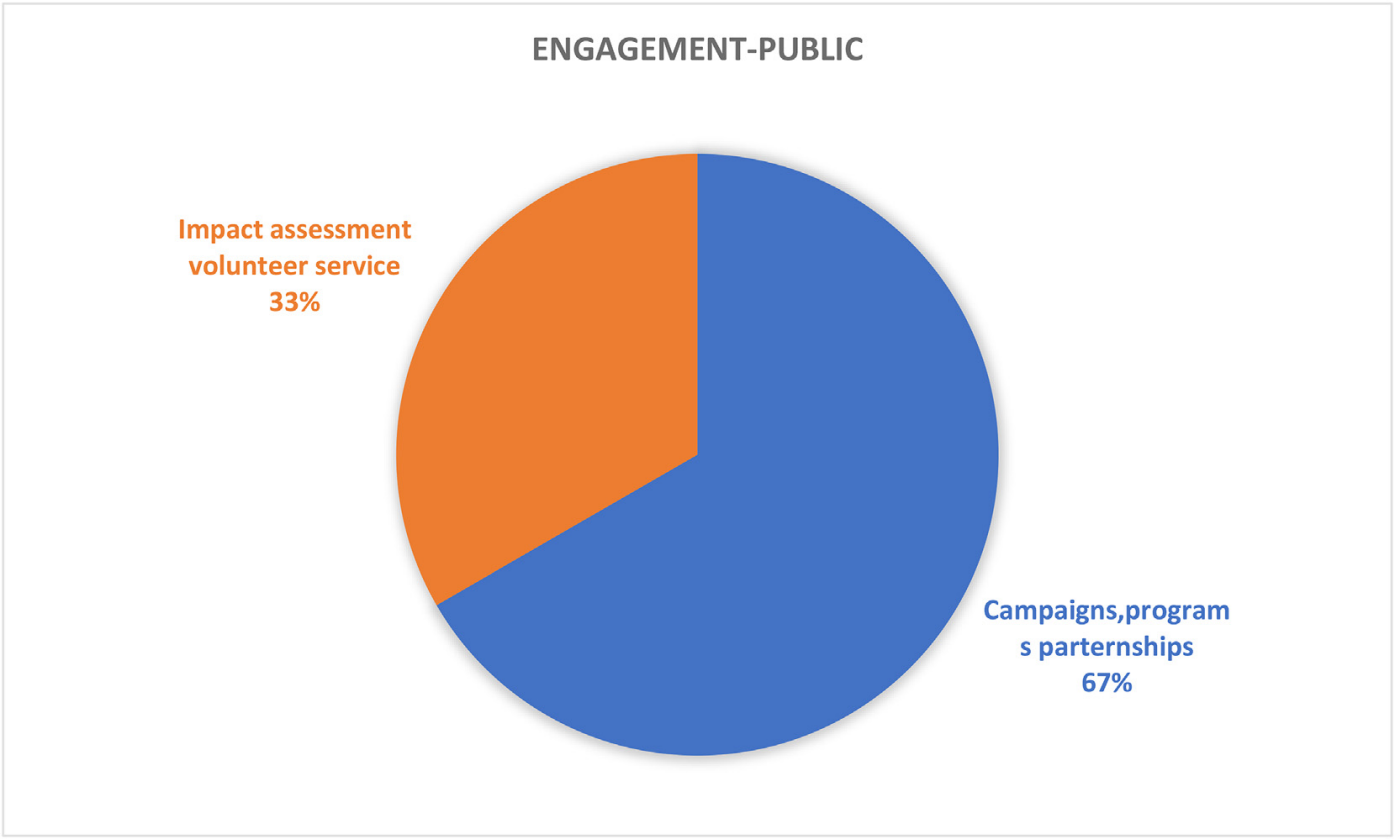
Coverage on the topic of engagement-public.

According to AASHE (the Association for the Advancement of Sustainability in Higher Education) (n.d.) public engagement refers to “*Engagement in community problem-solving is fundamental to sustainability. By engaging with community members and organizations in the governmental, non-profit, and for-profit sectors, institutions can help solve sustainability challenges. Community engagement can help students develop leadership skills while deepening their understandings of practical, real-world problems and the process of creating solutions*.” For the engagement-public aspect, there are only two parts involved in the literature: campaigns, programs partnerships (67%), and impact assessment volunteer service (33%) (see Figure 25). As seen, the most common forms of public engagement are campaigns, programs, and partnerships. Impact assessment volunteer services involves task-based engagements such as undertaking an impact assessment. Also, this kind of engagement require more knowledge and skills which may reduce people’s enthusiasm and willingness to partake. A major constraint of public engagement is how to get more participants. Regardless, public engagement provides several advantages such as allowing more stakeholders to get involved in and make more inclusive efforts towards campus sustainability, providing reflections and implications from different perspectives thus strengthening the implementation and acceptance of sustainability strategies and solutions. Therefore, it is suggested to employ a middle ground or “social entrepreneurs” when combining top-down and bottom-up approaches together. These social entrepreneurs conduct and support practical solutions to social, economic environmental and even institutional challenges when progress is hindered by systematic failures (Mohamad et al., 2018). The results reveal that engagement is still a relatively weak point in campus sustainability development. The main concern is how to improve the enthusiasm of students to participate in campus sustainability activities while getting various stakeholders from the community involved in campus sustainable development.

#### 3.3.12 Survey dimension (38 papers)

**Figure 26:**
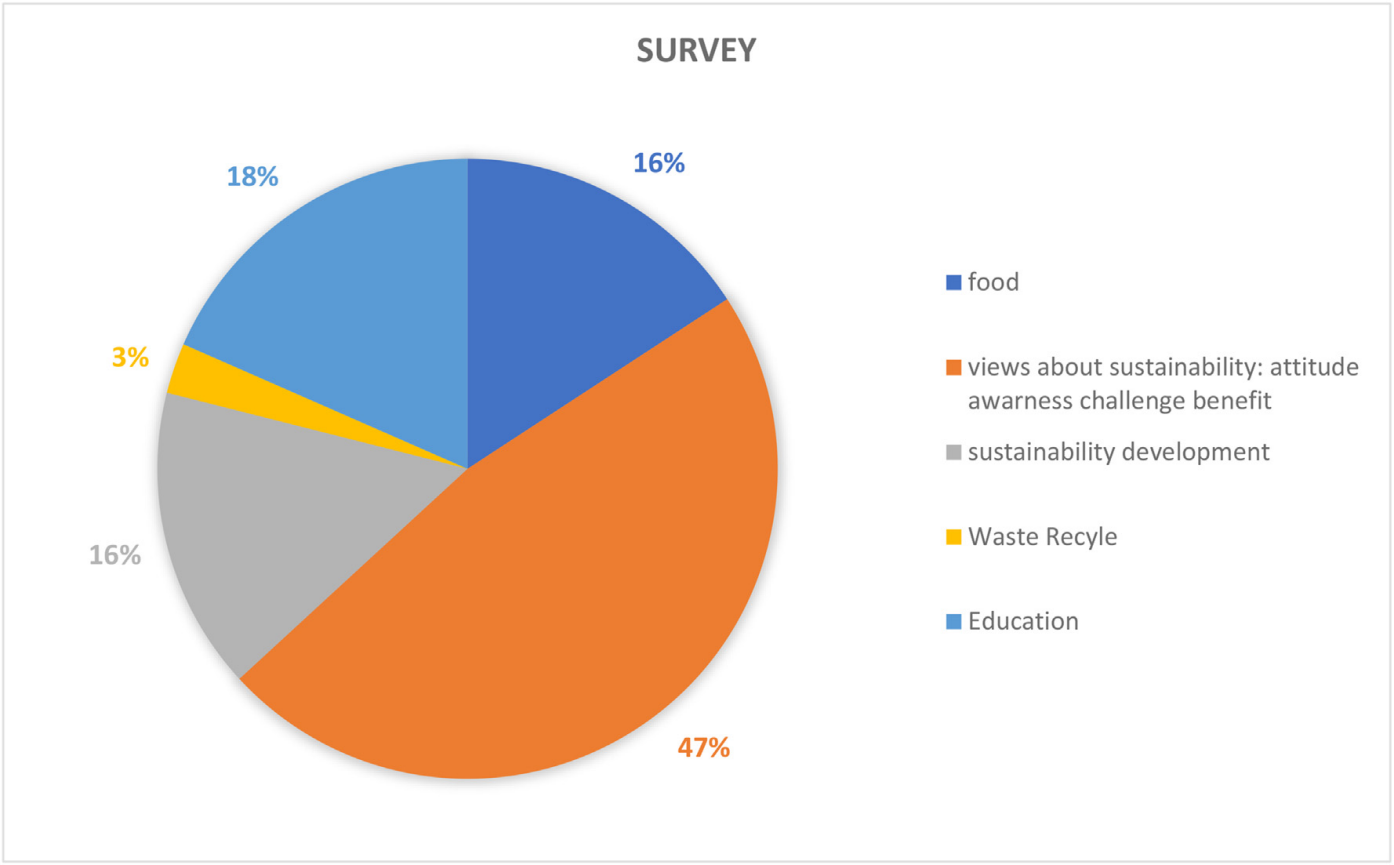
Coverage on the topic of survey.

Figure 26 depicts a limited coverage on the survey dimension. Many research results are based on the investigation, and the observation of sustainability attitude, awareness, challenges, and benefits (47%), implying the importance of consciousness when investigating human behaviour with reference to sustainability. The willingness and sustainability consciousness can influence the validity, accuracy, quality, and effectiveness of the survey. In some circumstances, low willingness of participants can lead to direct failure of conducting a survey, which limits the accuracy of results and subsequent recommendations. Other aspects related to the topic of survey include education (18%), food (16%), sustainability development (16%), and waste recycle (3%). Taking the previous 11 topics into account, these results reflect that campus sustainability studies do not conduct surveys on or may not have a lot of interests in the fields of governance, campus operation, building, transportation, and sustainability research. It also provides additional perspectives for enriching future CSAT development by adding elements/indicators related to food, waste recycling etc.

## 4 Conclusion and recommendations

This study developed a systematic literature review for CSATs covering 1194 articles and evaluation criteria and extracted 15 existing CSATs to provide an in-depth analysis on campus sustainability research and its implication to the dimensions of sustainability for emerging campus assessment tool. In total, this study considered different 12 dimensions, including governance dimension, operations-environmental, water, waste, buildings, transportation, operations-social, operations-financial, education dimension, research dimension, engagement campus and survey.

The most popular topic of CSATs is operations-environmental (30%), followed by education dimension (17%) and governance (9%). The focus of CSATs on operations-environmental is associated with energy consumption, greenhouse gases emissions, and energy efficiency, highlighting the critical role of sustainable energy use in CSATs for HEIs. Secondly, it is observed that internal and external policies are the main consideration in the governance dimension. Yet, there is a huge gap between extensive consideration and practical implementation of such policies. More homogeneity was found in the consideration of the water dimension which covers recycling strategy (32%), consumption (30%) and protection measures (28%) of water resources. Likewise, CSATs literatures show similar considerations in the waste dimension with waste system design, construction, renovation (34%), wastes system maintenance operation (33%), and hazardous waste (22%).

In the building dimension, strategies on architecture itself account for 46%, emphasizing its importance in campus sustainability as the physical carrier of sustainable education. Other elements in this dimension are related to green or environmentally friendly consideration, such as green office, green lab, etc. These green spaces are known for creating intelligent, personalized, and adaptive learning environment that can reduce the cognitive burden of students. Another benefit is the significant reduction in energy consumption that can be attained from buildings, and the fact that campuses are not only a physical workplace for employers, but also a symbolic cultural and social environment. For transportation, vehicle and public transportation circulation design are considered most in this category, implying the importance of campus design and infrastructures in supporting sustainable transportation of HEIs. It is suggested that campus design should encourage more non-motorized traffic, increase access to public transport options and implement more policies related to reducing the use of personal vehicles.

Within the social aspect of operations, CSATs articles were focused on working and living circumstances, human rights of staff and students and social and environmental responsibility. Evidence points to a need of a more balanced consideration under the three aspects mentioned. It can be suggested that future research should delve into some of the overlooked aspects with particular emphasis on handicapped design, employee satisfaction, recruitment, and training of staff on sustainability criteria, policy contribution remediation and disaster prevention within campuses. Conversely, the financial aspect of operations for CSATs (sustainability development investment), mostly considered economic performance, highlighting the concerns on balancing the cost and benefits of sustainable development investment among HEIs. Additionally, the education dimension is dominated by students’ sustainability education (91%) and only 9% of CSATs covered staff sustainability training. Since fulfilling SDGs of HEIs campus requires all stakeholders’ efforts, it is suggested that plans and courses in HEI should cover more extending areas and unconventional issues in sustainability. On the other hand, most CSATs on research dimension topics covered sustainable research (94%) while 6% of them are contributing to campus, community, and global SD.

Engagement-campus was only covered by 20 articles, the articles revealed the lack of consideration for stakeholders and/or public engagement during the establishment or development of initiatives that lead to sustainable campuses. Finally, the multiple benefits of surveys for providing various points of views, collecting feedbacks, and improving stakeholders’ engagements was examined. It was observed that many research results are based on the investigation, and the observation of sustainability attitude, awareness, challenges, and benefits (47%), implying the importance of consciousness when investigating human behaviour with reference to sustainability. The willingness and sustainability consciousness can influence the validity, accuracy, quality, and effectiveness of the survey. In some circumstances, low willingness of participants can lead to direct failure of conducting a survey, which may affect the inclusive implementation of a specific initiative, technology, or policy on campus. Willingness to participate and techniques to enhance survey both as an educational topic and also technique used on campus decision making is essential to sustainability drive in higher education.

For future development of CSATs, it is suggested that there is a need to address sustainability from a more balanced perspective as such CSATs should not only lean predominantly towards the environmental elements. This was the error discovered by other iterations of assessment tools such as NSATs and BSATs. Great improvement has been made over the years to balance these dimensions and as such CSATs should take up similar approach. Results also showed that there is no one size fit all solutions, since the dimensions of sustainability may vary based on the local context of the university under investigation. However, key topics should not be excluded all together. Hence topics of campus-engagement, survey approach, operations-financial (sustainable development investment) and building sustainability need to be considered thoroughly. More specifically CSATs should ensure that their categories and indicators cover topics such as campus-public engagement, survey on sustainable education, local development investment as well as more environmental elements such as building material, sustainable energy use, potable water, indoor environment, green buildings and slow traffic. Also, a strong case is to be made for the inclusion of smart campus systems through digital and automated innovation, which was visibly limited in the review of CSATs and the 1194 articles reviewed. This balancing act will ensure that users of CSAT have been giving more holistic option to consider when tackling campus sustainable design and development. Table 4 provides a breakdown of the results and recommendations of enhancing campus research and well as improving on the campus sustainability assessment tools.

## Declarations

### Author contribution statement

All authors listed have significantly contributed to the development and the writing of this article.

### Funding statement

Dr Ayotunde was supported by and part of a 10.13039/501100001809Natural Science Foundation of China (NSFC) funded project [72050410358].

### Data availability statement

The authors are unable or have chosen not to specify which data has been used.

### Declaration of interest’s statement

The authors declare no conflict of interest.

### Additional information

No additional information is available for this paper.

## References

[bib3] Alghamdi A., Hu G., Haider H., Hewage K., Sadiq R. (2020). Benchmarking of water, energy, and carbon flows in academic buildings: a fuzzy clustering approach. Sustainability.

[bib4] Alharthi A.D., Spichkova M., Hamilton M. (2019). Sustainability requirements for eLearning systems: a systematic literature review and analysis. Requir. Eng..

[bib5] Almufadi F.A., Irfan M.A. (2016). Initial estimate of the carbon footprint of Qassim university, Saudi Arabia. Int. J. Appl. Eng. Res..

[bib6] Alrashed S. (2020). Key performance indicators for smart campus and microgrid. Sustain. Cities Soc..

[bib7] Alshuwaikhat H.M., Abubakar I. (2008). An integrated approach to achieving campus sustainability: assessment of the current campus environmental management practices. J. Clean. Prod..

[bib9] Amaral L., Martins L., Gouveia J. (2015). Quest for a sustainable university: a review”. Int. J. Sustain. High Educ..

[bib10] Amey L., Plummer R., Pickering G. (2020). Website communications for campus sustainability: an analysis of Canadian universities. Int. J. Sustain. High Educ..

[bib11] Anwar N. (2020). Green Human Resource Management for organisational citizenship behaviour towards the environment and environmental performance on a university campus. J. Clean. Prod..

[bib12] Bayas Aldaz C.E. (2020). Understanding the university-sustainability link through media: a Spanish perspective. Sustainability.

[bib13] Boer P., Caeiro S., Walter L., Charbel J., Azeiteiro U. (2013). Sustainability Assessment Tools in Higher Education Institutions: Mapping Trends and Good Practices Around the World.

[bib14] Bugallo-Rodríguez A., Vega-Marcote P. (2020). Circular economy, sustainability and teacher training in a higher education institution. Int. J. Sustain. High Educ..

[bib15] Caeiro S., Walter L., Charbel J., Azeiteiro U. (2013).

[bib16] Calder W., Clugston R.M. (2003). International efforts to promote higher education for sustainable development”. Plann. High. Educ..

[bib17] Cattaneo M. (2018). Students’ mobility attitudes and sustainable transport mode choice. Int. J. Sustain. High Educ..

[bib18] Cebrián G., Palau R., Mogas J. (2020). The smart classroom as a means to the development of ESD methodologies. Sustainability.

[bib19] Chakraborty A. (2021). Building sustainable societies through purpose-driven universities: a case study from ashoka university (India). Sustainability.

[bib20] Clark C.R., Capps T.M. (2020). Synergy of the (campus) commons: integrating campus-based team projects in an introductory sustainability course. Sustainability.

[bib21] Cole L. (2003).

[bib22] Dalal-Clayton B., Bass S. (2002).

[bib100] Dawodu A., Akinwolemiwa B., Cheshmehzangi A. (2017). A conceptual re-visualization of the adoption and utilization of the Pillars of Sustainability in the development of Neighbourhood Sustainability Assessment Tools. Sustain. Cities Soc.

[bib101] Dawodu A., Cheshmehzangi A., Sharifi A., Oladejo J. (2022). Neighborhood sustainability assessment tools: Research trends and forecast for the built environment. Sustain. Futur..

[bib23] Dawodu A., Oladejo J., Tsiga Z., Kanengoni T., Cheshmehzangi A. (2022). Underutilization of waste as a resource: bottom-up approach to waste management and its energy implications in Lagos, Nigeria. Intell. Build. Int..

[bib24] Di Gerio C., Fiorani G., Paciullo G. (2020). Fostering sustainable development and social responsibility in higher education: the case of tor vergata university of rome. Manag. Dynamics Knowledge Eco..

[bib25] Disterheft A., Caeiro S.S., Ramos M.R., Azeiteiro U.M. (2012). Environmental management systems (EMS) implementation processes and practices in European higher education institutions: top-down versus participatory approaches”. J. Clean. Prod..

[bib26] Disterheft A., Caeiro S.S., Azeiteiro U.M., Leal Filho W., Caeiro S., Walter L., Charbel J., Azeiteiro U. (2013). Sustainability Assessment Tools in Higher Education Institutions: Mapping Trends and Good Practices Around the World.

[bib27] Er A.C., Nawi N.F.M., Tee M.Y., Ibrahim N.I., Bachok N. (2019). Entrepreneurial recycling initiatives towards campus sustainability. Int. J. Bus. Soc..

[bib28] Erbiyik Hikmet, Çatal Tuğçe, Durukan Sinem, Güneş Topaloğlu Doğan, Ünver Ümit (2021).

[bib30] Fraga-Lamas P. (2019). Design and experimental validation of a lorawan fog computing based architecture for iot enabled smart campus applications. Sensors.

[bib1] Ghani Abdul, Ahmad Bashawir, Nor Idayu Mahat, Hussain Azham, Mokhtar Mohd, Sanuri Sany (2019). Water sustainability in campus: a framework in optimizing social cost. Int. J. Recent Technol. Eng..

[bib31] Gómez F., Sáez-Navarrete C., Lioi S., Marzuca V. (2014). Adaptable model for assessing sustainability in higher education”. J. Clean. Prod..

[bib32] Grant K., Goldizen F.C., Sly P.D., Brune M.N., Neira M., van den Berg M., Norman R.E. (2013). Health consequences of exposure to e-waste: a systematic review. Lancet Global Health.

[bib33] Griffiths S. (2019). Exploring bluetooth beacon use cases in teaching and learning: increasing the sustainability of physical learning spaces. Sustainability.

[bib34] Hashim R.G., Md Sharif M.S., Muhammad R., Aminuddin Z.M. (2020). Sustainable campus income generation initiative and social entrepreneurship at a public university. Environ.-Behav. Proc. J..

[bib35] Hernandez-Escobedo Q. (2020). Sustainable solar energy in Mexican universities. Case study: the national school of higher studies juriquilla (UNAM). Sustainability.

[bib37] Jarillo M.P. (2019). Challenges of online higher education in the face of the sustainability objectives of the united nations: carbon footprint, accessibility and social inclusion. Sustainability.

[bib38] Kamal A., Asmuss M. (2013). Benchmarking tools for assessing and tracking sustainability in higher education institutions: identifying an effective tool for University of Saskatchewan”. Int. J. Sustain. High Educ..

[bib39] Kaplan D.H. (2015). Transportation sustainability on a university campus. Int. J. Sustain. High Educ..

[bib40] Kermath B. (2007). Why go native? Landscaping for biodiversity and sustainability education. Int. J. Sustain. High Educ..

[bib41] Li Y., Yang L., He B., Zhao D. (2014). Green building in China: needs great promotion. Sustain. Cities Soc..

[bib42] Lidgren A., Rodhe H., Huisingh D. (2006). A systemic approach to incorporate sustainability into university courses and curricula”. J. Clean. Prod..

[bib43] Liu W., Ma L., Li Y., Abuduwaili J., Abdyzhapar uulu S. (2020). Heavy metals and related human health risk assessment for river waters in the issyk− kul basin, Kyrgyzstan, central asia. Int. J. Environ. Res. Publ. Health.

[bib46] Lozano R. (2006). A tool for a graphical assessment of sustainability in universities (GASU)”. J. Clear Prod..

[bib47] Lozano R. (2006). Incorporation and institutionalization of SD into universities: breaking through barriers to change”. J. Clean. Prod..

[bib48] Lozano R. (2009). https://gin.confex.com/gin/2009/webprogram/Paper2614.html.

[bib49] Lozano R. (2011). The state of sustainability reporting in universities”. Int. J. Sustain. High Educ..

[bib50] Lozano R., Lukman R., Lozano F., Huisingh D., Lambrechts W. (2013). Declarations for sustainability in higher education: becoming better leaders, through addressing the university system”. J. Clean. Prod..

[bib51] Lukman R. (2009). Towards greening a university campus: the case of the university of maribor, Slovenia. Resour. Conserv. Recycl..

[bib52] Mawonde A., Togo M. (2019). Implementation of SDGs at the university of South Africa. Int. J. Sustain. High Educ..

[bib53] McHugh Amani N. (2011). https://hdl.handle.net/10161/5023.

[bib54] McKenna K., Altringer L. (2021). Alternative transportation education: implementing an innovative module. Int. J. Sustain. High Educ..

[bib55] Min-Allah N., Alrashed S. (2020). Smart campus—a sketch. Sustain. Cities Soc..

[bib56] Mohamad Z.F., Abd Kadir S.N., Nasaruddin A., Sakai N., Zuki F.M., Hussein H. (2018). Heartware as a driver for campus sustainability: insights from an action-oriented exploratory case study. J. Clean. Prod..

[bib58] Newman L. (2006). Change, uncertainty, and futures of sustainable development. Futures.

[bib59] Norton R., Brix A., Brydon T., Davidian E., Dinse K., Vidyarthi S. (2007). Transforming the university campus into a sustainable community. Plann. High. Educ..

[bib60] Oxenswärdh A., Persson-Fischier U. (2020). Mapping master students' processes of problem solving and learning in groups in sustainability education. Sustainability.

[bib61] Páez A., Whalen K. (2010). Enjoyment of commute: a comparison of different transportation modes. Transport. Res. Pol. Pract..

[bib65] Pujol F.A., Tomás D. (2020). Introducing sustainability in a robotic engineering degree: a case study. Sustainability.

[bib66] Ramos T.B. (2009). Development of regional sustainability indicators and the role of academia in this process: the Portuguese practice”. J. Clean. Prod..

[bib67] Ramos T., Pires S.M., Caeiro S., Walter L., Charbel J., Azeiteiro U. (2013). Sustainability Assessment Tools in Higher Education Institutions: Mapping Trends and Good Practices Around the World.

[bib68] Ramos T.B., Caeiro S., Melo J.J. (2004). Environmental indicator frameworks to design and assess environmental monitoring programs”. Impact Assess. Proj. Apprais..

[bib70] Roorda N., Fillo W.L. (2002). Teaching Sustainability at Universities: towards Curriculum Greening.

[bib71] Roorda N., Caeiro S., Walter L., Charbel J., Azeiteiro U. (2013). Sustainability Assessment Tools in Higher Education Institutions: Mapping Trends and Good Practices Around the World.

[bib72] Rybarczyk G., Gallagher L. (2014). Measuring the potential for bicycling and walking at a metropolitan commuter university. J. Transport Geogr..

[bib73] Saldaña-Durán C.E., Messina-Fernández S.R. (2021). E-waste recycling assessment at university campus: a strategy toward sustainability. Environ. Dev. Sustain..

[bib74] Shealy T. (2016). Do sustainable buildings inspire more sustainable buildings?. Procedia Eng..

[bib75] Shriberg M. (2002). *Institutional* assessment tools for sustainability in higher education: strengths, weaknesses, and implications for practice and theory. Int. J. Sustain. High Educ..

[bib77] Silva L.C.C.da (2019). Water sustainability potential in a university building – case study. Sustain. Cities Soc..

[bib78] Simpson Walter. (2003).

[bib79] Smith H.M. (2018). Public responses to water reuse – understanding the evidence. J. Environ. Manag..

[bib80] Smyth D.P., Fredeen A.L., Booth A.L. (2010). Reducing solid waste in higher education: the first step towards ‘greening’ a university campus. Resour. Conserv. Recycl..

[bib81] Suwartha N., Sari R.F. (2013). Evaluating UI GreenMetric as a tool to support green universities development: assessment of the year 2011 ranking. J. Clean. Prod..

[bib82] Tangwanichagapong S. (2017). Greening of a campus through waste management initiatives: experience from a higher education institution in Thailand. Int. J. Sustain. High Educ..

[bib83] Terhell Su-Lin, Cai Kevin, Chiu Dylan, Murphy Johnathon (2015).

[bib86] Tiana S.A., Villarreal A.A. (2016). A collaborative programme in sustainability and social responsibility. Int. J. Sustain. High Educ..

[bib87] Too L., Bajracharya B. (2015). Sustainable campus: engaging the community in sustainability. Int. J. Sustain. High Educ..

[bib88] Tsai J.-H., Tang Y.-T. (2012). Design for Innovative Value towards a Sustainable Society.

[bib90] United Nations (2015). https://sdgs.un.org/goals.

[bib91] Velazquez L., Munguia N., Sanchez M. (2005). Deterring sustainability in higher education institutions: an appraisal of the factors which influence sustainability in higher education institutions”. Int. J. Sustain. High Educ..

[bib92] Velazquez L., Munguia N., Platt A., Taddei J. (2006). Sustainable university: what can be the matter?. J. Clean. Prod..

[bib94] White S.S. (2020). Student food insecurity and the social equity pillar of campus sustainability. Int. J. Sustain. High Educ..

[bib95] Wright T.S.A., Corcoran P.B., Wals A.E.J. (2004). Higher Education and the Challenge of Sustainability: Problematics, Promise, and Practice.

[bib96] Xiong W., Mok K.H. (2020). Sustainability practices of higher education institutions in Hong Kong: a case study of a sustainable campus consortium. Sustainability.

[bib98] Zhou J. (2016). Proactive sustainable university transportation: marginal effects, intrinsic values, and university students' mode choice. Int. J. Sust. Transport..

[bib99] Zint M. (2016). Campus sustainability and social sciences. Int. J. Sustain. High Educ..

